# A novel broad-spectrum antibacterial and anti-malarial *Anopheles gambiae* Cecropin promotes microbial clearance during pupation

**DOI:** 10.1371/journal.ppat.1012652

**Published:** 2024-10-23

**Authors:** Cairé Barreto, Victor Cardoso-Jaime, George Dimopoulos

**Affiliations:** W. Harry Feinstone Department of Molecular Microbiology and Immunology, Bloomberg School of Public Health, Johns Hopkins University, Baltimore, Maryland, United States of America; Oregon State University, UNITED STATES OF AMERICA

## Abstract

Anophelinae mosquitoes are exposed to a variety of microbes including *Plasmodium* parasites that cause malaria. When infected, mosquitoes mount versatile immune responses, including the production of antimicrobial peptides. Cecropins are one of the most widely distributed families of antimicrobial peptides in insects and all previously studied *Anopheles* members are playing roles in adult mosquito immunity. We have identified and characterized a novel member of the *Anopheles gambiae* cecropin family, cecropin D (CecD), that is uniquely expressed and immune-responsive at late larval stages to promote microbial clearance through its broad-spectrum antibacterial activity during larval-pupal developmental transition. Interestingly, Cecropin D also exhibited highly potent activity against *Plasmodium falciparum* sporozoites, the malaria parasite stage that is transmitted from mosquitoes and infects humans and thereby holds promise as a malaria transmission-blocking agent. Finally, we have defined unequivocal cecropin-specific molecular signatures to systematically organize the diversity of the cecropin family in malaria vectors.

## Author summary

*Anopheles* mosquitoes that transmit the deadly malaria are exposed to a variety of microbes in their natural habitats. Mosquitoes use their innate immune system that also comprises antimicrobial peptides to fight infections with these microbes including the *Plasmodium* malaria parasite. Cecropins are one of the most widely distributed antimicrobial peptides in insects and all previously studied *Anopheles* cecropins are playing roles in adult mosquito immunity. We have identified and characterized a novel *Anopheles gambiae* cecropin, cecropin D (CecD), that is uniquely produced and immune-responsive at late larval stages to promote broad spectrum microbial clearance during larval-pupal developmental transition. Interestingly, Cecropin D also shows potent activity against *Plasmodium falciparum* sporozoites, the malaria parasite stage that is transmitted from mosquitoes to humans and could therefore be developed into a malaria control strategy.

## Introduction

Malaria remains a significant global health concern, with *Anopheles gambiae* serving as a primary vector for the transmission of the *Plasmodium* parasite. In 2022, 249 million cases were reported globally, resulting in over 600,000 deaths. Most of these deaths were attributed to *Plasmodium falciparum*, the major causative agent of human malaria [1]. Efforts to combat this scourge will require the development of novel control strategies involving the blocking of *Plasmodium’s* infection cycle within the mosquito vector by targeting any of its developmental stages prior to transmission to a vertebrate host [2].

Upon infection, mosquitoes assemble a robust immune response that combines both cellular and humoral effectors [3]. These reactions, primarily mediated by the Toll and Imd signaling pathways, can also target *Plasmodium* and cause major parasite losses [4]. Within the arsenal of mosquito humoral immunity, antimicrobial peptides (AMPs) are key players and serve as frontline defenders against invading pathogens. Classically, AMPs are defined as small cationic peptides (<10 kDa) that organize into a secondary structure in which hydrophobic and hydrophilic amino acids are spatially segregated to create an amphiphilic overall nature, which allows them to both diffuse in aqueous environments and penetrate into a lipid milieu, such as cellular membranes [5]. As a result, AMPs exhibit strong antimicrobial properties by disrupting bacterial cell membranes or interfering with vital intracellular processes [6–9].

The discovery of insect AMPs over 40 years ago marked a significant milestone in efforts to comprehend the molecular basis of invertebrate immunity and shed light on the key role antibacterial effectors play in invertebrate defense mechanisms, especially in species of economic importance. Since then, numerous insect AMPs have been identified and characterized, providing a solid body of knowledge on the fundamental biology of immune mechanisms in lower animals [10–16]. Moreover, with the growing concern regarding the spread of recalcitrant infectious diseases caused by microbes resistant to conventional treatments, including bacterial and parasite strains, there has been a resurgence of interest in exploring the diversity of insect AMPs, which are envisioned as promising candidates for a new generation of drugs to tackle the global antimicrobial resistance crisis [17]. Most of the current knowledge concerning the molecular mechanisms underlying insect innate immune responses has been provided by studies in the fruit fly *Drosophila melanogaster*, which encodes at least seven AMP families with distinct mechanisms of action and antimicrobial specificities [18]. Conversely, two AMP families represent the major contributors to antimicrobial immunity in mosquitoes: the defensins and the cecropins. However, different mosquito species may have additional AMPs with single members each, such as gambicins [4] and the *in-silico* predicted attacins [19] of the malaria mosquito *An*. *gambiae*.

Cecropins, originally identified in the hemolymph of immune-activated pupae of the giant silk moth *Hyalophora cecropia*, represent one of the most abundant families of AMPs in insects and hold the distinction of being the first-ever reported AMPs in invertebrates [20]. Since their initial identification, cecropins (and cecropin analogs such as sacrotoxins, stomoxins, papiliocins, enbocins, and spodopsins) have been discovered in numerous insect species, spanning the orders Lepidoptera, Coleoptera and Diptera [21–23]. Consistent with other gene families, cecropins are usually found as multigenic families, consisting of both functional and non-functional genes (pseudogenes). In the silkworm *Bombyx mori*, 12 cecropin genes were identified and divided in subtypes (A, B, D, E and Enbocins) [24]. Similarly, the cecropin locus of *H*. *cecropia* contains three cecropin genes encoding the functional Cec A, B and D [25]. Among Diptera, genomic analysis of *D*. *melanogaster* revealed the existence of a tight cecropin cluster comprising three functional cecropin genes (Cecropins A1, A2 and B) interspaced by two pseudogenes (Cecψ1 and Cecψ2) along with a fourth functional cecropin C located ~3-kb upstream cecropin B gene [23,26]. In coleopteran insects, cecropin genes have been identified in several beetle species such as *Acalolepta luxuriosa* [27], *Paederus dermatitis* [28], *Oxysternon conspicillatum* [22] and *Calomera littoralis* [29].

Cecropins are short (3–5 kDa) linear α-helical, highly cationic peptides characterized by an N-terminal hydrophilic helical region linked to a hydrophobic C-terminus by a short hinge region. These compounds display a wide spectrum of lytic activities, targeting both Gram-negative and Gram-positive bacteria as well as filamentous fungi, yeasts, and some protozoans [30]. Mechanistically, cecropins are known to inactivate bacterial cells by membrane disruption through pore formation [31,32]. Most insect cecropins are amidated at their C-terminus and harbor a tryptophan at position 1 or 2 of the mature peptide. However, mosquito cecropins may not follow this conserved structural pattern [33–35].

Cecropins are key immune factors of antipathogen defenses, and their relevance also extends to biotechnological applications since they offer promise in addressing modern biomedical challenges such as anti-tumoral therapies, antimicrobial resistance, and the development of novel malaria control methods [30]. Indeed, insect cecropins and cecropin analogs have been shown to inhibit a multitude of drug-resistant bacteria, such as *Pseudomonas aeruginosa* and *Acinetobacter baumanni*, currently listed by WHO as of critical priority and requiring the urgent development of new treatment strategies [36,37]. In addition, cecropins have shown activity against the opportunistic pathogen *Candida albicans*, can suppress the proliferation of human immunodeficiency virus 1 (HIV), and also block protozoan parasites such as *Trypanosoma cruzi* and *P*. *falciparum* [33,38,39].

Despite the functional versatility and broad range of potential applications of cecropins, surprisingly few studies have reported their presence in mosquito vectors of human infectious diseases [33–35,40]. Of the approximately 460 species of *Anopheles* reported to date (of which 30 to 40 are known vectors of human malaria), studies on the antimicrobial or antiparasitic spectrum of cecropins are restricted to one cecropin of *An*. *gambiae*, AngCecA [33]. The chemically synthesized peptide has been shown to display antibacterial activity towards a panel of Gram-negative and Gram-positive bacteria, filamentous fungi, and yeasts. Two additional cecropin-coding genes (i.e., cecropin B and cecropin C) have been described and are located in the so-called “cecropin cluster” on the X chromosome under the control of promoter regions regulated by NF-κB (CecA and CecB) or dorsal (CecC) transcription factors [41]. The overall lack of studies on *Anopheles* cecropins has led to misleading nomenclature and confusion regarding the true extent of cecropin diversity in mosquitoes. We conducted *in-silico* explorations for cecropin sequences across publicly accessible databases leading to the serendipitous identification of a fourth member of the *An*. *gambiae* cecropin family, previously reported by Christophides and colleagues as CEC4 [19] but left wholly uncharacterized. In the present work, this cecropin has been designated as Cecropin D (CecD) to align with previously established nomenclature [33,41] and has been recognized as a promising candidate for further investigation because of its potential involvement in host-microbe interactions and anti-*Plasmodium* activity.

## Results

### Cecropin D, a novel member of cecropin family in *An*. *gambiae*, displays unique features and chromosomal location while conserving gene architecture

**Fig 1:**
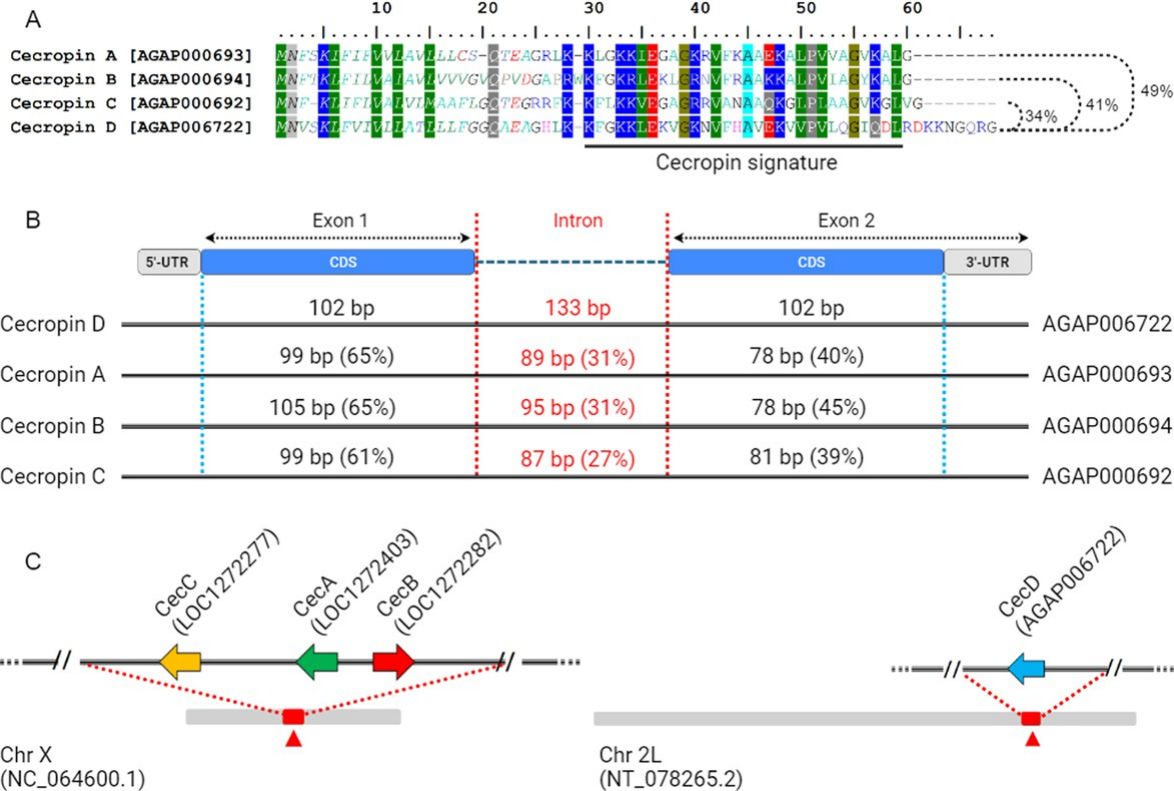
Sequence comparison, gene organization and genomic location of *Anopheles gambiae* cecropins. (A) Comparison of the amino acid sequences of the four precursor cecropins of *An*. *gambiae*. Conserved amino acid residues are highlighted. Percentage numbers to the right represent pairwise sequence identity of cecropin D relative to its paralog precursors. The ruler indicates the relative position of each residue. (B) A not-to-scale schematic representation of gene architecture of the four cecropin genes found in *An*. *gambiae*. Lengths and nucleotide sequence identities of intronic sequences and exonic coding sequences (CDS) relative to the cecropin D gene are indicated. (C) Scheme illustrating the topological distribution of cecropin coding genes across *An*. *gambiae* chromosomes.

By means of a comprehensive *in silico* approach, we have characterized a fourth member of the cecropin family in the malaria vector *An*. *gambiae*. This cecropin has been tentatively named Cecropin D (CecD) to follow the initial nomenclature of *An*. *gambiae* cecropins, which includes Cecropins A, B, and C. CecD full-length cDNA sequence consisted of a 5’-terminal untranslated region (UTR) of 70 bp, a 3’-UTR of 151 bp, and an open reading frame (ORF) of 204 bp encoding a precursor peptide of 67 amino acids, starting with a predicted 24-residue signal sequence followed by a highly cationic mature peptide of 43 amino acids (**S1 Fig**). Sequence alignment of cecropin precursors showed that Cecropin D shares the highest identity with cecropin A (49%), followed by cecropin B (41%) and cecropin C (34%). Interestingly, the mature forms of cecropins A, B, and C showed higher amino acidic identities when compared to each other, indicating a greater sequence distance between those cecropins and cecropin D (**Fig 1A** and **S1 Table**). Also, cecropin D displayed a longer mature peptide, which is attributed to the presence of an additional 9-residue cationic C-terminal tail (**Fig 1A**). The molecular parameters of all the *An*. *gambiae* cecropins are summarized in **S2 Fig.** Furthermore, the prediction model of Cecropin D structure reveals that the molecule adopts a three-dimensional arrangement nearly identical to previously described cecropins, consisting of two α-helices separated by a flexible hinge region (**S3 Fig**).

Like its paralog cecropins A-C, cecropin D is encoded by a single gene which shares a conserved organization consisting of two exons separated by a single intron (**Fig 1B**). The first exon spans 172 bp, encompassing the 5’-UTR and a 102-bp region that encodes the signal peptide and the first 10 amino acid residues of the mature peptide. The second exon, spanning 253 bp, encodes the residual peptide sequence, including the cecropin domain, and the 3’-UTR (**S1 File**). Pairwise nucleotide sequence comparison revealed that exon 1 of cecropin D exhibits higher sequence identity with cecropins A, B, and C (65%, 53%, and 61% respectively), than does exon 2, which shares 40% identity with cecropin A, 45% identity with cecropin B, and 39% identity with cecropin C (**Fig 1B** and **S1 Table**). The analysis of topological organization of cecropins genes in mosquito genome revealed that Cecropin D gene is located outside the cecropin cluster on autosomal chromosome 2, in contrast to cecropins A, B, and C, which have been shown to be clustered midway on the X chromosome (**Fig 1C**).

### Cecropin family in malaria vectors is diverse but not uniformly distributed among species

**Fig 2:**
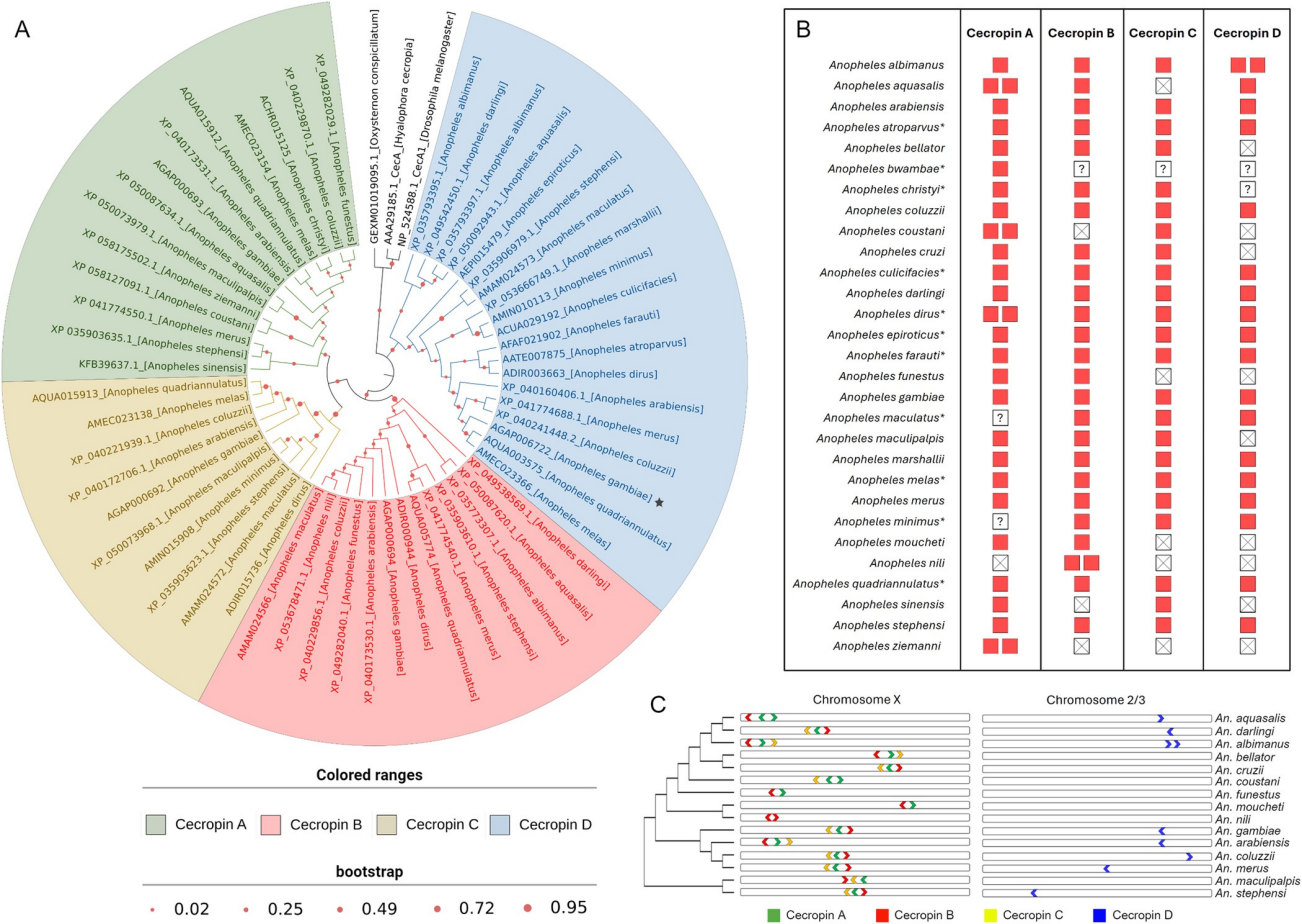
Phylogenetic analysis, repertoire and chromosomal distribution of cecropin genes in Anophelinae mosquitoes. (A) Maximum-likelihood phylogenetic tree generated from mature peptide sequences of *Anopheles* cecropins showing the existence of four discrete cecropin clades, each represented by a cecropin member. The dark star indicates the cecropin D sequence from *An*. *gambiae*. (B) Distribution of cecropin genes across the 29 species of *Anopheles* mosquitoes studied. Available mosquito genome-deduced proteomes were scanned to identify cecropin-coding genes. Red boxes indicate the presence of one gene copy, while empty boxes represent its absence. Asterisks denote species for which cecropin sequences were retrieved solely from Blast searches because of the unavailability of proteomes, and question marks (?) indicate cases in which the absence of cecropin genes cannot be ascertained because of insufficient data. (C) Not-to-scale chromosomal distribution of cecropin genes in *Anopheles* species. Cecropin genes were mapped onto their respective chromosomes, depicted by chevron arrows indicating gene orientation. The left-side cladogram represents the phylogenetic relationship between mosquito species based on neighbor-joining of 18S rDNA nucleotide sequences.

To explore the evolutionary relationship between CecD and other mosquito cecropins, and to confirm its identity as a true member of the cecropin family, a maximum-likelihood phylogenetic analysis was performed, including 101 cecropin sequences from 29 mosquito species. The resulting phylogenetic tree revealed that cecropins of Anophelinae mosquitoes are monophyletic and encompasses four distinct members representing the described cecropins A, B, C, alongside a fourth group including Cecropin D. Furthermore, the analysis suggests that the diversification of the cecropin family in *Anopheles* mosquitoes began with an initial gene duplication, leading to the formation of cecropin D and the ancestor gene of cecropins A/B/C, followed by a duplication event that produced cecropin B and the common ancestor of cecropins A and C, which further diverged through another duplication, resulting in cecropins A and C. (**Fig 2A**). Notably, the cecropin D clade was composed of 19 sequences, of which 18 represented orthologous sequences of *An*. *gambiae* cecropin D retrieved from 17 mosquito species, suggesting that cecropin D emerged early in the ancestral lineage of the Anophelinae subfamily. Additionally, the identification of four discrete groups allowed us to define group-specific molecular signatures that improve their classification in mosquitoes. These molecular signatures are proposed here to address the misleading nomenclature commonly found across publicly available databases and clearly associate a given cecropin sequence to a specific group. To establish unique amino acidic signatures, sequences from each group were systematically aligned and amino acid residues common to all sequences were assigned as part of the conserved signature (**S4 Fig** and **S2 File**). Group-specific molecular signatures within mature peptides of Anophelinae cecropins were defined as follows:

Cecropin A: GXLKKLGKKXEX_2_GXRVFXAXEKXLPVX_4_KALG;
Cecropin B: APRX_[0,1]_WKFGKRLEXLGRNVFXAAXKALPVX_2_GYKAX_[0,1]_LG;
Cecropin C: X_2_FXKXLX_5_GRRX_3_AAQKX_2_P;
Cecropin D: GXLX_2_GKKLEKXGX_2_VX_3_EXVVX_3_.

Our extensive cecropin dataset also provided insights into the dynamics shaping cecropin evolution within the Anophelinae subfamily. Among the 29 mosquito species examined, 15 harbored the complete set of cecropins (A-D), while 10 species lacked cecropin members. Four species (e.g., *Anopheles bwambae*, *Anopheles christyi*, *Anopheles maculatus*, and *Anopheles minimus*), also appeared to lack at least one cecropin member, although definitive conclusions require further validation due to the absence of genome-deduced proteomes for these species. Gene duplication events were common, particularly for cecropin A, which showed the highest prevalence in four out of six species. Cecropin B had two copies exclusively in *Anopheles nili*, while no duplication of cecropin C was observed. Cecropin D exhibited the highest gene deletions and was duplicated only in *Anopheles albimanus* (**Fig 2B and S3 File**). The topological arrangement of cecropin genes within chromosomes showed a consistent pattern across mosquito species, mirroring that of *An*. *gambiae*, with genes A, B, and C clustered on the X chromosome and cecropin D located on autosomal chromosome 2 or 3. Consistent syntenic organization of cecropin coding genes was observed across species, although in several cases the cecropin cluster was not located in the same chromosomal region. For instance, while cecropins A-C were positioned approximately midway along the X chromosome in *An*. *gambiae*, *An*. *maculipalpis*, and *An*. *stephensi*, the cecropin cluster in *An*. *aquasalis* and *An*. *albimanus* occupied the extremities of the X chromosome. Similarly, the location of the cecropin D gene varied among species, although in all species it is found solitary in a distinct chromosome (**Fig 2C and S3 File**).

### Cecropin D is a late larval infection responsive broad-spectrum antimicrobial peptide that mediates bacteria clearance prior to pupation

**Fig 3:**
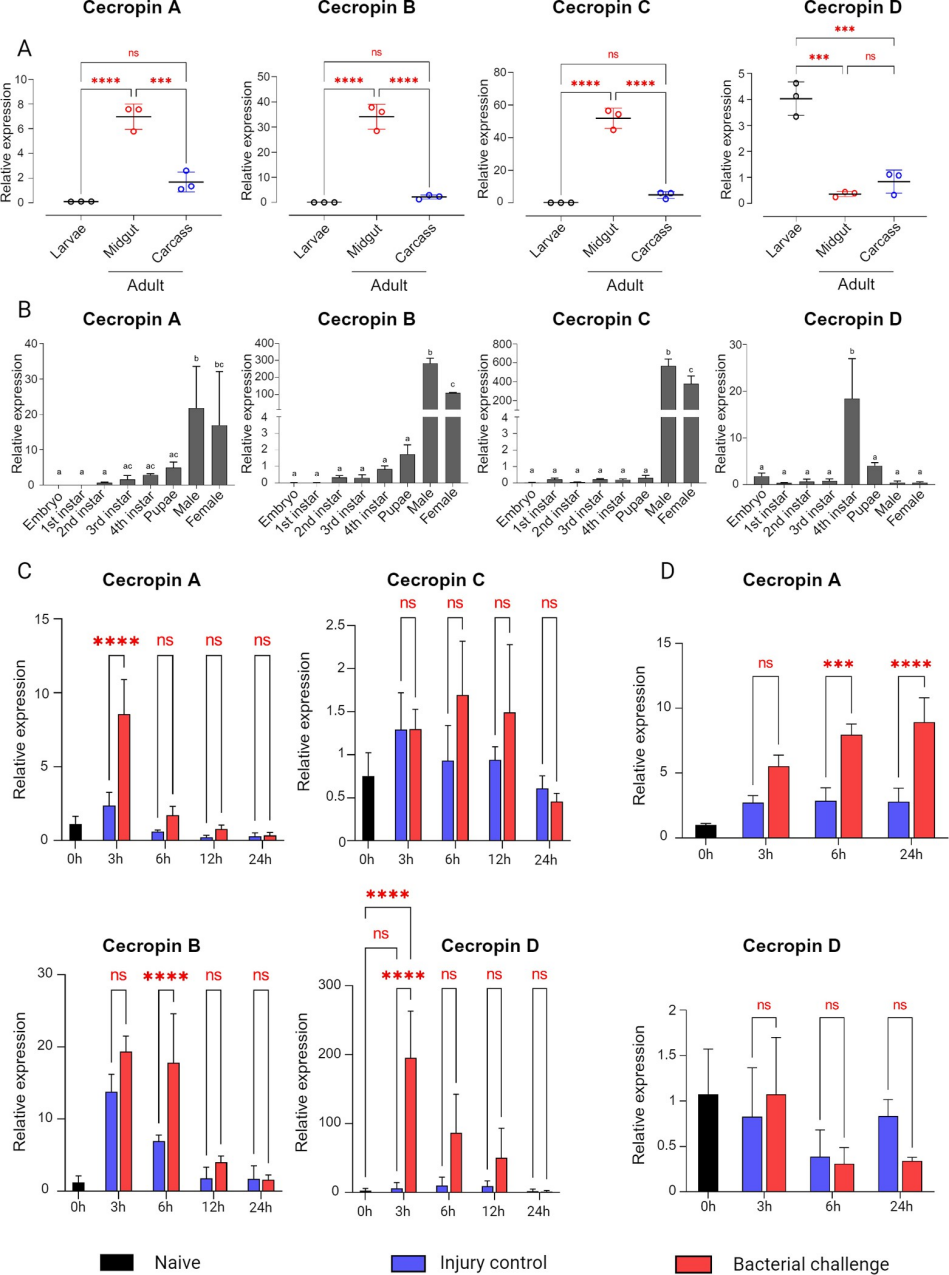
Spatial-temporal transcript distribution of cecropin genes in naïve *An*. *gambiae* mosquitoes and transcriptional response to bacterial stimulation. (A) Transcript distribution of cecropin genes in larvae and adults. A pool of larval instars (L1-L4), together with midguts and carcasses of adult mosquitoes, were collected, and relative gene expression was determined by RT-qPCR. Data were normalized to the average Ct values of all samples, and *An*. *gambiae* RpS7 was used as an internal control. Data are shown as the mean of three biological replicates ± SD. Statistical analysis: One-way ANOVA followed by Tukey’s multiple comparison test. ns: not significantly different. ***, *p* = 0.0001; ****, *p* < 0.0001. (B) Relative transcript abundance of cecropins during larval development and in adults. Data were normalized to the average Ct values of all samples, and *An*. *gambiae* RpS7 was used as an internal control. Data are shown as the mean of four biological replicates ± SD. Statistical analysis: One-way ANOVA followed by Tukey’s multiple comparison test. Different letters indicate statistical difference. (C) Transcriptional response of cecropin genes after larval bacterial injections. Larvae at the fourth instar stage were cold-anesthetized and injected with a heat-killed suspension of *E*. *coli* and *S*. *aureus*. Non-manipulated larvae and larvae injected with filter-sterile PBS were used as controls. Data were normalized to the average Ct values of naïve larvae, and *An*. *gambiae* RpS7 was used as an internal control. Data are shown as the mean of four biological replicates ± SD. Statistical analysis: One-way ANOVA followed by Tukey’s multiple comparison test. ns: not significantly different. ****, *p* < 0.0001. (D) Transcriptional response of cecropin A and cecropin D upon adult bacterial stimulation. Five-day-old adult females were cold-anesthetized and injected with a heat-killed bacterial suspension. Non-manipulated adults and adults injected with filter-sterile PBS were used as controls. Data were normalized to the average Ct values of naïve adults, and *An*. *gambiae* RpS7 was used as an internal control. Data are shown as the mean of four biological replicates ± SD. Statistical analysis: One-way ANOVA followed by Tukey’s multiple comparison test. ns: not significantly different. ***, *p* = 0.0001; ****, *p* < 0.0001.

To uncover the potential role of cecropin D in Anophelinae mosquitoes, we used *An*. *gambiae* as a model to investigate its transcriptional profile at an organismal level and compared it with previously described cecropins A-C. We analyzed gene expression in terms of spatial-temporal transcript abundance and the transcriptional response upon systemic bacterial challenge. Developmental expression of the CecD gene was analyzed across all larval instar stages, and adult tissue-specific mRNA abundance was assayed in the midguts and carcasses of naïve 5-day-old adult females. Cecropins A-C were primarily expressed in adult midguts, with negligible expression at larval stages and in adult carcasses. Cecropin C had the highest midgut mRNA abundance relative to larvae and carcasses, followed by cecropin B and cecropin A. In contrast, cecropin D was primarily present at larval stages (**Fig 3A**). Expression dynamics throughout mosquito development corroborated cecropins A-C predominantly in adults, whereas cecropin D peaked in the fourth instar larval stage prior to pupation (**Fig 3B**). We further investigated the transcriptional response of cecropin genes following systemic bacterial stimulation in the fourth larval instar. Naïve larvae were injected with heat-inactivated Gram-negative *E*. *coli* and Gram-positive *S*. *aureus*, and cecropin expression levels were quantified using RT-qPCR. This challenge increased mRNA abundance of cecropins A, B, and D shortly after injection, with cecropin D showing a 32-fold rise at 3 hours post-injection (hpi) compared to the PBS-injected control, then returning to basal levels within 24 hours. Cecropin A had sustained high expression at 3 and 6 hpi, declining by 12 and 24 hpi, while cecropin B showed significant upregulation at 3 hpi, declining at 6 hpi, with lower sustained levels at 12 and 24 hpi. Cecropin C showed no response to bacterial injections (**Fig 3C**). Interestingly, in adult mosquitoes, cecropin A increased at 6 and 12 hpi with bacterial suspension, whereas larval-specific cecropin D showed no response, suggesting that pathways controlling expression of cecropin D in larvae differ from those controlling expression of paralogs CecA-C in adults (**Fig 3D**).

**Fig 4:**
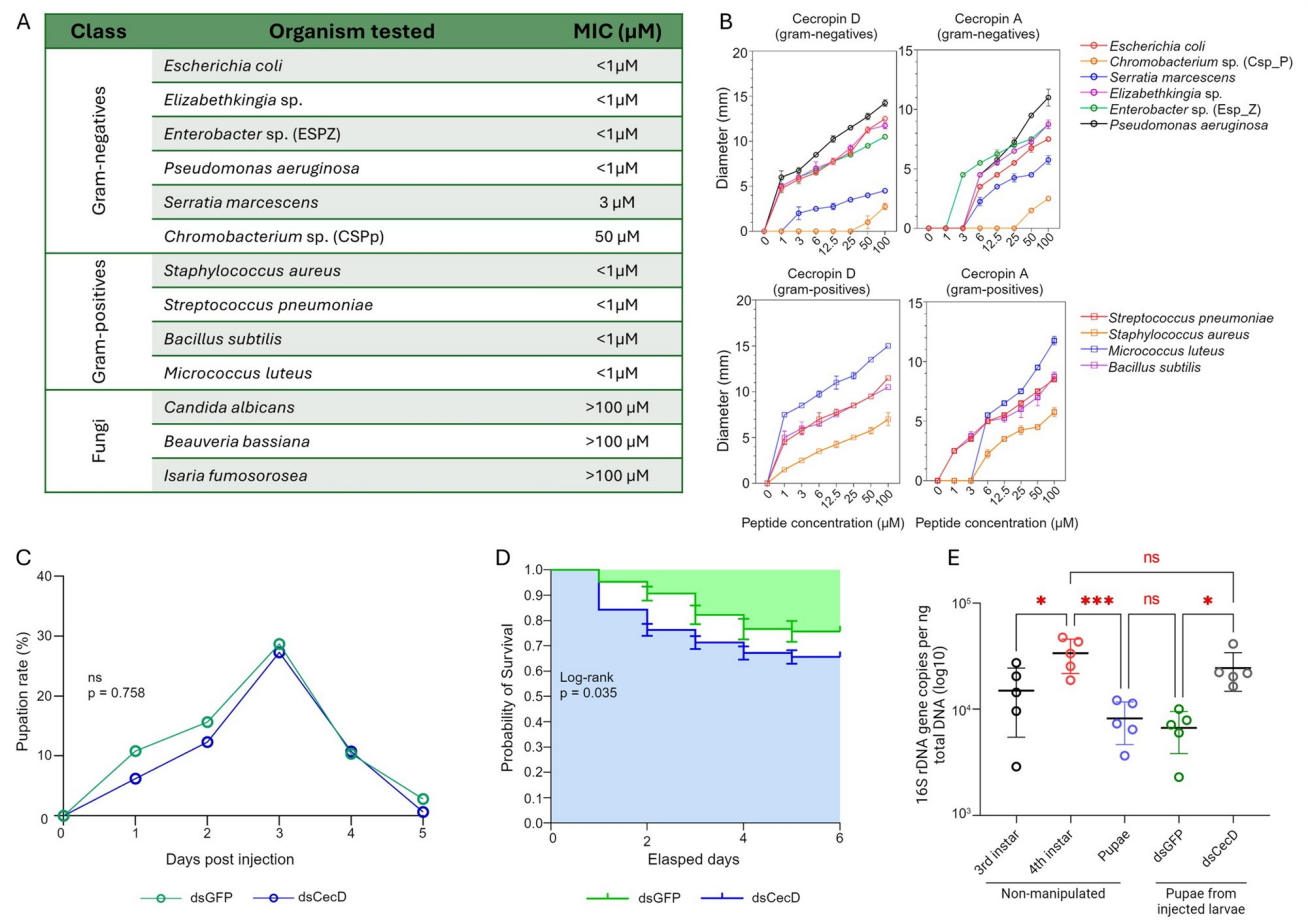
Antimicrobial activity of synthetic cecropin D and effects of RNAi-mediated cecropin D knock-down on mosquito larvae. (A) Summary of the minimum inhibitory concentrations (MICs) of cecropin D against bacteria and fungi. The MIC (μM) was determined by the radial diffusion method. Synthetic cecropin D peptide was loaded at 1-, 3-, 6-, 12.5-, 25-, 50- and 100 μM into 3-mm wells on agar plates containing viable bacteria, and allowed to diffuse for 3 hours. Antimicrobial activity was determined as a function of the presence and size of inhibitory zones. The MIC represents the minimum concentration at which an inhibitory zone can be detected. (B) Comparison between the inhibitory zones of cecropin D of *An*. *gambiae* and cecropin A of *H*. *cecropia*. Bacteria were exposed to synthetic peptides, and the diameter of the inhibitory zones was measured. (C) Pupation rate of silenced larvae. Fourth instars were injected with either cecropin D (dsCecD) or dsGFP as a control, and pupation was monitored. Pupation rates were normalized to the number of dead larvae and expressed as the percentage of total living larvae on a given day. (D) Survival proportions of silenced larvae. Viability of injected larvae was monitored for 6 days, and the number of dead larvae was recorded daily. Survival curves for the dsCecD- and dsGFP-injected larvae were compared with a log-rank Mantel-Cox analysis. (E) Quantification of total bacterial load of naïve and injected insects. Naïve third (L3) and fourth (L4) instars as well as naïve pupae and pupae derived from injected larvae were collected and subjected to total DNA extraction for absolute quantification of bacterial 16S rDNA. Data are presented as the average number of 16S copies per ng of total DNA. Each dot represents a pool of five individuals. Statistical analysis: One-way ANOVA followed by Tukey’s multiple comparison test. ns: not significantly different. *, *p* < 0.05; **, *p* < 0.001.

To investigate the antibacterial activity of cecropin D, the synthetic peptide was tested against a panel of Gram-negative and Gram-positive bacteria, two entomopathogenic fungi (*Isaria fumosorosea and Beauveria bassiana*), and the yeast *Candida albicans* using radial diffusion assays. Cecropin D peptide demonstrated a broad-spectrum antibacterial activity (**Fig 4A**). Among the Gram-negative strains tested, *Escherichia coli* W3110, *Elizabethkingia* sp., *Enterobacter* sp., and *Pseudomonas aeruginosa* exhibited significant susceptibility to cecropin D, as evidenced by observable zones of growth inhibition, even at the lowest tested concentration (1 μM). Notably, *P*. *aeruginosa* displayed the largest zones of inhibition. Conversely, *Serratia marcescens* and *Chromobacterium* sp. showed limited susceptibility, with the latter being resistant to peptide up to a 25 μM concentration. Cecropin D also exhibited activity against all tested Gram-positive bacteria (i.e., *Staphylococcus aureus*, *Streptococcus pneumoniae*, *Micrococcus luteus*, and *Bacillus subtilis*), generating clear inhibitory zones at concentrations up to 1 μM, with *Micrococcus luteus* displaying the largest zones of inhibition (**Fig 4B and S4 File**). No inhibition of fungal growth was detected at any of the tested concentrations.

Due to its potent *in vitro* antibacterial activity, RNAi-mediated gene silencing was used to study cecropin D functions *in vivo* during late larval development. Fourth instar larvae were injected with double-stranded RNA targeting cecropin D or green fluorescent protein (GFP) as a control. At 24 hrs after dsRNA injection, Cecropin D silencing efficiency was 97% whereas the expression levels of cecropins A, B, and C remained unaffected (**S5 Fig**). Silencing of cecropin D did not affect pupation, which peaked three days after dsRNA injections in both the cecropin D dsRNA- and GFP dsRNA-injected groups, but it reduced the viability of the larvae by about 10% (**Fig 4C and 4D**). To address whether such decreased viability was attributable to bacterial proliferation, bacterial load in pupae from surviving cecropin D-silenced larvae was measured by quantifying 16S rDNA and compared to GFP control pupae. The baseline bacterial load of naïve larvae was similarly assessed by including non-injected larvae (third and fourth instars) and pupae controls. A notable increase in microbiota was observed as larvae progressed from the third to fourth instar, likely due to gut expansion, followed by a decrease during pupation. Pupae from GFP controls had bacterial loads similar to non-injected pupae, while cecropin D-silenced pupae had a 3.6-fold higher bacterial load, suggesting a significant contribution of cecropin D to bacterial clearance during late larval development (**Fig 4E**).

### Cecropin D inhibits *Plasmodium falciparum* salivary gland-stage sporozoites *in vitro*

**Fig 5:**
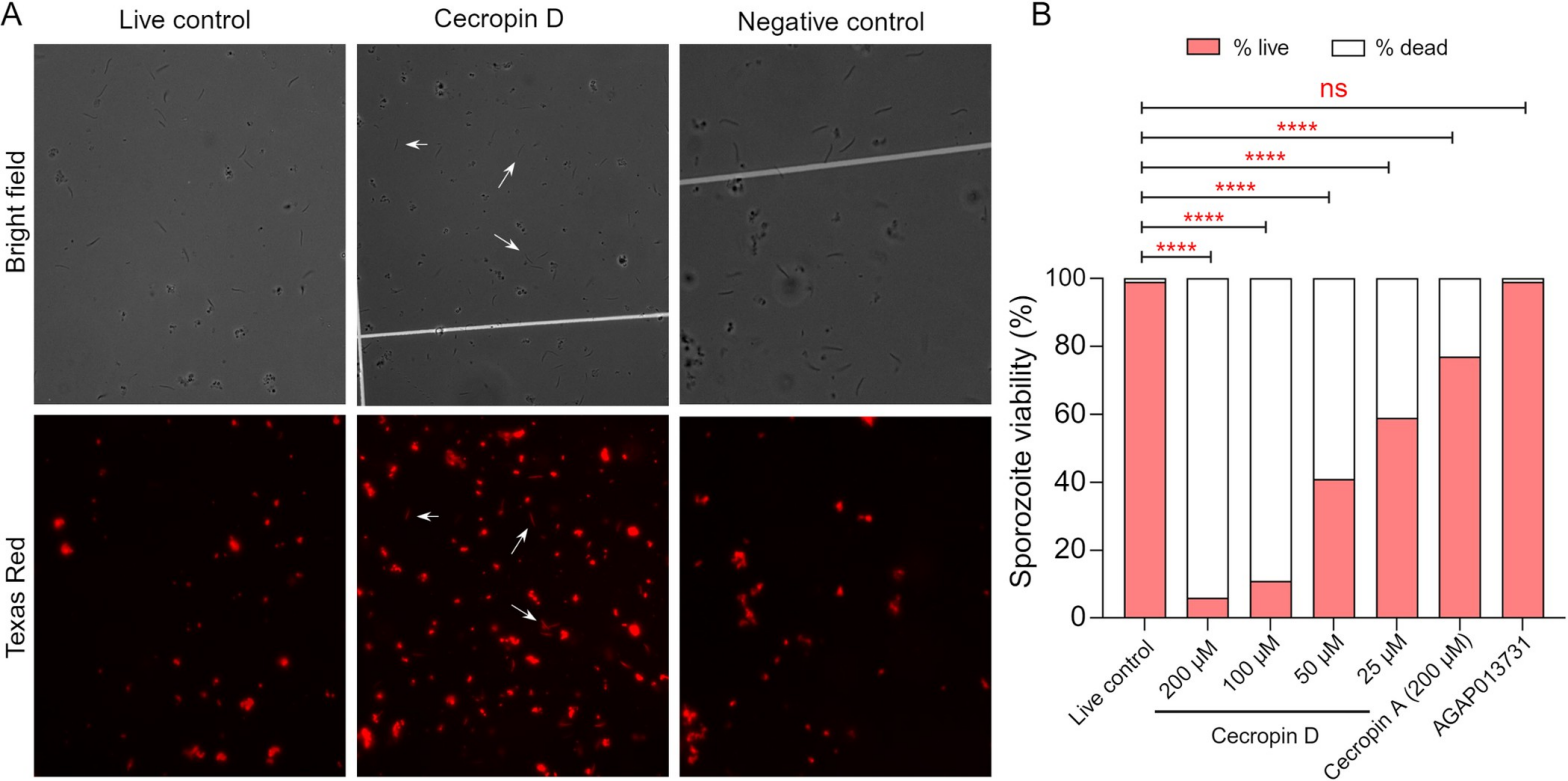
Anti-*Plasmodium* activity of synthetic cecropin D. (A) Viability of *P*. *falciparum* sporozoites after incubation with cecropin D peptide. Sporozoites were collected from infected salivary glands, and parasites were incubated with a 200-μM concentration of the synthetic cecropin D peptide. Sporozoites incubated with either sterile PBS or the innocuous synthetic peptide AGAP013731 were used as controls. Parasites were incubated with fluorescent viability markers and counted under the microscope. Arrows indicate dead *P*. *falciparum* sporozoites in both bright-field and with the Texas Red fluorescence filter. Dead sporozoites appear as glowing-red cells. Scale: 10 μm. (B) Viability of *P*. *falciparum* sporozoites upon incubation with cecropin D at vanishing concentrations. Sporozoites were isolated from infected salivary glands and incubated with 200-, 100-, 50- or 25 μM of cecropin D peptide. The lepidopteran cecropin A (200 μM) was included to assess whether anti-sporozoite activity is a universal trait of insect cecropins. Sporozoites were incubated with fluorescent viability markers, and viability was determined as the number of non-viable glowing-red parasites relative to the total number of sporozoites counted under bright field. Fisher’s exact test was used to test for significance. ns: not significantly different. ****, *p* < 0.0001.

To investigate the ability of cecropin D to inhibit the sporogonic stages of malaria parasites, we incubated synthetic peptides with *P*. *falciparum* sporozoites and assessed cell viability using fluorescence microscopy with live/dead fluorescent markers. Initially, antiparasitic activity was tested on freshly isolated sporozoites (~100,000 cells) exposed to a 200 μM peptide solution. Sporozoites incubated with sterile PBS served as a control for cell viability, and the peptide AGAP013731, produced alongside cecropin D but without detectable antimicrobial activity, was used as a control for possible toxicity of synthetic peptides. Exposure to cecropin D, but not to PBS or synthetic peptide controls, resulted in increased red fluorescence in sporozoites, indicating decreased parasite viability (**Fig 5A**). Given the non-physiological concentration, we tested cecropin D at lower concentrations to assess dose dependence. Approximately 100,000 freshly isolated sporozoites were exposed to serially diluted cecropin D ranging from 200 μM to 25 μM, and viability was measured after one hour. Cecropin A (Sigma-Aldrich) from *H*. *cecropia* served as a control to determine if anti-*Plasmodium* activity was universal to insect cecropins or specific to *A*. *gambiae* cecropin D. Cecropin D consistently reduced sporozoite viability compared to PBS and synthetic peptide controls, showing a dose-response relationship; parasite viability increased as peptide concentration decreased. At 200 μM, 93.6% of sporozoites exhibited red fluorescence associated with cell damage, 89% at 100 μM, 58.5% at 50 μM, and 41% at 25 μM (Fig 5B). Notably, 200 μM of synthetic cecropin A also significantly reduced sporozoite viability, with approximately 23% showing cell damage (**Fig 5B**). However, cecropin D at the same concentration led to a substantially higher proportion of cell death, suggesting that the larval-specific cecropin D is more potent than cecropin A against *P*. *falciparum* sporozoites.

## Discussion

Insects have flourished across diverse ecosystems largely due to their efficient defense systems equipped with a variety of immune mechanisms capable of recognizing and neutralizing invading microorganisms. Particularly, insect AMPs hold significant promise in the current post-antibiotic era, as they efficiently combat bacteria through diverse mechanisms without inducing significant resistance [17]. Among these peptides, cecropins represent strong candidates for addressing modern biomedical challenges, such as malaria control. Christophides and colleagues [19] previously proposed a CEC1-4 nomenclature for cecropin genes in the malaria vector *An*. *gambiae*, although cecropins were first described as Cecropins A-C [33,41]. Here, in agreement with a historical point of view, these cecropins were referred to as Cecropins A-C being synonyms of CEC1-3, and their naming can be used interchangeably. Through a comprehensive *in-silico* approach, we have characterized a cecropin in the principal vector of human malaria in sub-Saharan Africa *An*. *gambiae*, tentatively named cecropin D (previously referred to as CEC4). The Cecropin D sequence emerged from a combinatory search method which involved retrieving unidentified candidate sequences from the *An*. *gambiae* genome with potential antimicrobial properties, guided by conserved sequence features of AMPs, and further filtering using AMP predictor software.

Cecropin D of *An*. *gambiae* encodes a 67-amino acid precursor, which undergoes processing to yield a mature 43 residue peptide after cleavage of a 24-residue N-terminal signal sequence. Cecropin D features a longer mature peptide with a unique highly cationic C-terminal tail, establishing it as the most divergent among *An*. *gambiae* cecropins. Cecropins typically exhibit an N-terminal amphipathic and cationic domain linked to a hydrophobic C-terminal domain by a short hinge region, facilitating their antimicrobial mechanism by adopting an α-helical structure upon contact with bacterial membranes [32,42]. CecD distinct length and composition suggest potential adaptations or specialized functions, possibly in response to specific microbial challenges. The exon 1 of cecropin D shares a higher sequence identity with the corresponding exon of its paralogs cecropins A-C than exon 2, encoding the entire signal peptide and the amino-terminal cationic region crucial for membrane interaction. On the other hand, the hydrophobic C-terminal domain, encoded in exon 2, is thought to mediate killing by interaction with acyl chains and insertion into lipid bilayers [31]. We speculate that, because no specific amino acid composition may be mandated aside from imparting a hydrophobic nature, the C-terminal region of cecropins may exhibit lower evolutionary stringency with regards to the presence of specific amino acid residues. The reason for our speculation is that a multitude of amino acid residues can confer a hydrophobic nature, whereas cationic residues are comparatively limited, potentially accounting for the higher sequence identity at the N-terminus as a result of positive selection. However, further investigation into the molecular evolution of cecropin genes of *Anopheles* mosquitoes, including analyses of the ratio of non-synonymous to synonymous substitutions along both exons, is imperative to corroborate this hypothesis.

The mature Cecropin D peptide is thought to consist of two helices separated by a flexible hinge region, similar to other insect cecropins. The six lysine residues present at the N-terminus give it a highly cationic nature, which likely enhances its antimicrobial activity. Studies of the structure-function relationship of synthetic cecropin peptides have demonstrated the critical role of the cationic N-terminus in bacterial inhibition [43]. Additionally, the Val-Pro sequence in Cecropin D’s hinge region, which separates the two α-helices, may be crucial for providing the flexibility needed for cecropin action. Synthetic peptides lacking this flexible region have shown significantly reduced antibacterial activity compared to their more flexible analogs [44]. Akin other mosquito cecropins, Cecropin D lacks a tryptophan residue at position 2. It has been suggested that this Trp2 residue, which is conserved in all *H*. *cecropia* cecropins, is essential for the first interaction with bacterial membranes. Replacement of Trp2 with the helix-forming non-aromatic glutamic acid drastically reduces cecropin A antibacterial activity [45]. The strong antibacterial properties of Cecropin D indicate that Trp2 is not essential for its function, which may suggest that the initial interaction of mosquito cecropins with bacterial membranes may be mediated by a distinct mechanism from that observed in other insect cecropins. Lastly, we draw attention to the additional cationic tail in Cecropin D, which consists of nine extra residues, including four cationic amino acids linked to the hydrophobic C-terminal α-helix. Despite having a hydrophilic region connected to its hydrophobic C-terminus, Cecropin D still retains its antibacterial activity. Thus, the effect of this unique structural feature on Cecropin D function remains to be fully explored.

Cecropin D are broadly found within multiple species of *Anopheles*, including the Neotropical species *An*. *darlingi* and *An*. *albimanus*, known to have firstly diverged in Anophelinae lineage more than 100 million years ago [46]. Such a broad distribution suggests an early emergence, likely due to gene duplication events, as a conserved gene architecture was observed among *An*. *gambiae* paralogs. Gene duplication has been widely recognized as a key mechanism driving the evolution of cecropins in insects. Analysis of the silkworm *Bombyx mori* genome sequence revealed evidence of gene duplication, supported by the presence of transposable elements in the 5’ and 3’ flanking regions of each paralogous gene [24]. Phylogenetic studies of cecropin genes from *B*. *mori* and *Spodoptera exigua* further suggest that these genes share a common ancestral origin but evolved independently in each species through successive duplication events [47]. In the *Drosophila* genus, cecropin genes are typically found in multiple copies that are closely clustered as neighboring genes and often accompanied by pseudogenes [48]. These features, combined with the presence of both highly divergent and highly similar gene copies within a species has led some researchers to propose that the cecropin gene family in *Drosophila* evolves according to Nei’s birth-and-death model [49]. This model suggests that gene duplication is followed by the preservation or loss of copies, with minimal selective pressure for diversification. Given the broad-spectrum, non-target-specific nature of cecropins as antimicrobial peptides, it is speculated that natural selection primarily favors maintaining an optimal number of functional gene copies to ensure an effective antibacterial response, rather than driving site-specific modifications in the peptide’s amino acid composition.

In contrast, our phylogenetic analysis suggests that the expansion of the cecropin gene family in the Anophelinae lineage predates speciation and has evolved under positive selection. The molecular diversity of the cecropin family in malaria vectors encompasses four members: cecropins A-D and most species studied harbor all cecropin members, exhibiting consistent sequence variation. According to this model, multiple gene duplication events in a common ancestor of *Anopheles* led to the emergence of cecropin D, which was the first to diverge, followed by the divergence of cecropin B, and finally, a duplication event that gave rise to cecropins A and C. The clustering of cecropin sequences by gene group rather than by species, along with evidence that cecropin genes have been maintained in Anophelinae genomes for an extended period further supports this hypothesis. Such long-term retention and diversification of cecropin genes suggest that they have been subject to selective pressures favoring functional diversification. However, we do not exclude the possibility of birth-and-death evolutionary forces acting on Anophelinae cecropin genes, as instances of gene duplications and deletions (or possibly pseudogenization) at a species-specific level have been observed in certain species, such as *An*. *coustani*, *An*. *nili*, *An*. *ziemanni* and *An*. *albimanus*. Hence, these forces may act synergistically, contributing to the dynamic evolution of cecropins in the *Anopheles* genus. In addition, such arrangement enabled the recovery of group-specific molecular signatures based on conserved amino acids, providing means to systematically organize and classify cecropin diversity in mosquitoes with a view toward proposing an unambiguous nomenclature and mitigate confusion in both literature and public databases. We propose a universal naming system for Anophelinae cecropins which include: (i) species identification using a six-letter system in italics (three for the genus and three for the species) followed by a space, (ii) the cecropin group identified as CecX followed by a period, and (iii) a number for the identification inside the group in case multiple variants are identified. Thus, An. gambiae cecropins, including cecropin D described here, would be referred to as *Anogam* CecA-D.1.

Comparison of mosquito cecropins with those in other insects like *D*. *melanogaster* and *B*. *mori* underscores their greater molecular diversity and polymorphism, which is likely influenced by the varied immune challenges mosquitoes face during lifetime [23,24]. Mosquitoes engage in blood feeding and undergo larval development in microbial-rich environments, thereby experiencing diverse and unique selective pressures on their immune systems. Blood digestion results in a proliferation of bacterial communities in the gut, and larval development takes place in various aquatic habitats that can greatly differ with regard to their microbiomes [50,51]. These environmental factors likely contribute to the evolution of antimicrobial peptide diversification in Anophelinae mosquitoes. The sequence divergence of cecropin D may also be attributed to its genomic location since it does not reside within the cecropin cluster on the X chromosome in any of the species in which the gene was identified; instead, it is located on autosomal chromosome 2 or 3. This location across diverse mosquito species implies an ancient evolutionary event underlying the relocation of the cecropin gene, which may have significant implications for regulatory processes governing gene expression.

The predominant expression of cecropins A-C in adult mosquitoes, particularly within the midgut, is likely indicative of a role in maintaining healthy bacterial levels in the midgut tissue [52]. In contrast, the expression of cecropin D, a potent and broad-spectrum antibacterial peptide, at a stage prior to pupation may suggest a role in the bacterial clearance during larval-pupal transition, as it temporally correlates with a natural decrease in the endogenous larval microbiome. Indeed, a transcriptional profiling of larval gut sections have demonstrated a significant enrichment of antimicrobial peptides, including cecropin D, in the gastric caeca, where they presumably counteract the large microbial loads ingested by the larvae during course of development [53]. Therefore, the consistent reduction in larval bacterial loads observed in the present study is likely linked to a decrease in the gut-associated microbiome. Given the susceptibility of larvae to various microbial pathogens present in their aquatic habitats [54], the strong activation of cecropin D expression upon larval immune stimulation further supports this hypothesis. Cecropin D silencing did not impact pupation rates, which indicates a minimal direct involvement in developmental regulation. Instead, a slight increase in larval mortality upon cecropin D silencing was observed, further suggesting a role in antibacterial defenses along with other antibacterial effectors, such as larval-specific defensins identified in *An*. *gambiae* [54,55]. We also observed a significant reduction in bacterial loads during the transition from fourth instar larvae to pupa in non-manipulated insects, indicating that bacterial clearance may be critical for successful pupation. These findings align with previous studies on other mosquito species, such as *Anopheles punctipennis*, *Culex pipiens*, and *Aedes aegypti*, which reported reductions of 95%, 96%, and 86% in total bacterial load of fourth instars compared to pupae, respectively [56]. Accordingly, such reduction was no longer observed in those pupae derived from surviving cecropin D-silenced larvae, further corroborating the participation of cecropin D in bacterial clearance during preparation for metamorphosis.

Cecropin D displayed robust activity against both Gram-negative and Gram-positive bacteria. Activity against Gram-positive bacteria has been generally demonstrated for several cecropins, but the vast majority have shown mild to no activity toward *S*. *aureus*, which is noteworthy as a medically relevant bacterial species, given the growing emergence of multidrug resistance [30,57]. In our study, we observed antibacterial activity of *An*. *gambiae* cecropin D against both *S*. *aureus* and *Streptococcus pneumoniae*; the latter known to be a causative agent of pneumonia and meningitis [58]. Conversely, *Serratia marcescens* and *Chromobacterium* sp. exhibited lower sensitivity to cecropin D. Resistance to antibiotics and cationic antimicrobial peptides has been previously demonstrated in both nosocomial and environmental isolates of *S*. *marcescens* [59]. This resistant phenotype is often attributed to the widespread presence of efflux pumps that extrude toxic compounds, such as antibiotics, into the external environment, as well as to chemical modifications in surface lipopolysaccharides (LPS) that reduce negative net charge of cell surface and therefore diminish interaction with cationic antimicrobial peptides [60,61]. Such recalcitrance to naturally occurring AMPs may explain *Serratia* capability to rapidly and stably colonize diverse tissues in both male and female mosquitoes, a feature exploited to engineer recombinant bacteria expressing anti-*Plasmodium* effectors for use in paratransgenesis to combat malaria [62]. Similarly, *Chromobacterium violaceum* employs multidrug-resistance genes and drug exclusion translocases, enhancing resilience to endure unfavorable environmental conditions [63]. However, limited literature is currently available regarding the surface composition of *Chromobacterium*, including chemical modifications of LPS moieties that may account for the resistance phenotype observed here.

Cecropins typically undergo carboxy-terminal amidation, which is believed to enhance antimicrobial activity and peptide stability [64]. Our peptide sequence analysis revealed that all four *An*. *gambiae* cecropins are C-terminally glycine-extended, suggesting that they are prone to C-terminal amidation for production of fully active peptides. Amidation of cecropin A selectively improved activity against Gram-positive bacteria and blocked filamentous fungi and certain species of yeasts but did not uniformly enhance efficacy across all species [33]. In our study, no antifungal activity of cecropin D was observed. Nonetheless, due to the lack of experimental evidence regarding cecropin D amidation, we conducted our antimicrobial assays using glycine-extended synthetic peptides only. Therefore, cecropin D may exhibit extended antimicrobial spectrum, potentially including activity against filamentous fungi and yeasts.

In addition to its antimicrobial properties, cecropin D exhibits a remarkable *in vitro* activity against *P*. *falciparum* sporozoites, the stage of the parasite that is transmitted into the mammalian host from an infected mosquito. Early studies on insect cecropins, including synthetic derivatives of cecropin B from *H*. *cecropia*, like SB-37 and Shiva-1, demonstrated their ability to hinder *P*. *falciparum* asexual stages by disrupting essential nutrient uptake, thus affecting parasite viability [39]. Moreover, injections of the synthetic *H*. *cecropia* cecropin B in *An*. *gambiae* resulted in a notable reduction in oocyst development when challenged with *P*. *cynomolgi*, underscoring the potential of cecropins against malaria parasites [65]. However, studies have predominantly focused on lepidopteran cecropins, especially cecropin B from the cecropia moth. Diverging from this trend, Kim and colleagues [66] engineered *An*. *gambiae* to overexpress the endogenous cecropin A within mosquito gut using a blood-meal inducible promoter, resulting in a 60% reduction in *P*. *berghei* oocysts, while transgenic *Ae*. *aegypti* with similar induction system of endogenous cecropin A and defensin A showed refractoriness to *P*. *gallinaceum* [67]. Here, we demonstrate that synthetic cecropin D peptide effectively reduces *P*. *falciparum* sporozoite viability in a concentration-dependent manner. Even at the lowest concentration tested (25 μM), over 40% of the parasites exhibited reduced viability while the lepidopteran cecropin A would only kill significantly at 200 μM. *In vitro* anti-*Plasmodium* activity of insect cecropins typically require concentrations in the range of 50–100 μM [39,68,69]. However, those studies have primarily focused on blood-stage forms, so that the higher concentration requirements observed may be attributed to the reduced susceptibility of the chemically distinct nature of the parasite’s cell membrane, presumed to be the target for cecropin action [70]. Cecropin D specific expression at pre-adult stages suggests that it does not play an anti-*Plasmodium* role in malaria-transmitting wild-type mosquitoes, and the weak selective pressure that *Plasmodium* exerts on the mosquito, especially at the sporozoite stage, suggests that it did not evolve to specifically possess antiparasitic activity. However, the potential utility of *An*. *gambiae* cecropin D anti-sporozoite activity is interesting and worth further exploration.

The use of mosquito transgenic lines expressing anti-*Plasmodium* effectors, including antimicrobial peptides, has been extensively investigated and shown to be a promising malaria transmission-blocking strategy [71]. However, complete blocking in the midgut tissue remains elusive because of the parasite’s ability to evade engineered blocking mechanisms and ultimately colonize the salivary glands [72]. Therefore, a complementary strategy could involve the transgenic expression of cecropin D in mosquito salivary glands or the fat body to target the sporozoites during their egress from the oocysts, effectively eliminating the surviving parasites. Moreover, the presence of cecropin D as a naturally occurring peptide within the mosquito immune system allows for genetic engineering with minimal manipulation, as achieved by introducing an appropriate endogenous promoter to regulate gene expression at desired sites. Future research endeavors should focus on elucidating the mechanistic basis of Cecropin D anti-*Plasmodium* activity, including the identification of molecular targets and pathways involved in parasite inhibition.

## Materials and methods

### *In silico* gene discovery

Putative novel AMPs of *An*. *gambiae* were screened against VectorBase (https://vectorbase.org/vectorbase/app). Searches were focused on genes encoding short-chain (3–10 kDa), cationic (p*I* 7–14) peptides featuring a secretion signal sequence at the NH_2_-terminus. Three independent AMP prediction tools were employed (Antimicrobial Peptide Scanner vr.2 [73], iAMPpred [74], and AI4AMP [75]), and peptide sequences having a prediction probability ≥80% were systematically selected. Only candidates with consensus across all predictive models were retained. AMP candidates underwent further analysis for the presence of conserved structural domains utilizing the Conserved Domain Database (https://www.ncbi.nlm.nih.gov/Structure/cdd/wrpsb.cgi).

An uncharacterized *An*. *gambiae* sequence (VectorBase: AgaP_AGAP006722; GenBank: CM000356.1) harboring a cecropin-like motif served as a query in a Blast-based search for ortholog sequences in non-redundant protein datasets specific to “Anophelinae.” To expand the cecropin collection, *in-silico* data mining was extended to all genome-deduced proteomes of *Anopheles* currently available at NCBI. Cecropin amino acid sequences were aligned using MAFFT v.7 (https://mafft.cbrc.jp/alignment/server/), and the resulting alignment was utilized to establish a universal amino acid signature for the Anophelinae cecropin motif, defined as follows:

$$\left[\text{KR}\right]\text{X}\left(0,5\right)\text{GX}\left(2\right)\left[\text{VI}\right]\text{X}\left(5\right)\text{KX}\left(2\right)\text{PX}\left(3\right)\left[\text{GA}\right],$$

where X represents any amino acid residue, the numbers within parentheses (*n*, *m*) represent intervals from *n* to *m* residues, and the residues within square brackets [XY] indicate either X or Y.

This signature was applied as a probe to scan Anophelinae proteomes using the ScanProsite tool (https://prosite.expasy.org/scanprosite/) with default cut-off parameters.

### Molecular cloning and sequencing

PCR reactions were carried out in a 15-μL reaction volume containing 1 μL of cDNA, 0.1 μM of each primer, and 7.5 μL of OneTaq 2X Master Mix (New England Biolabs Inc.) Primer sequences are listed in **S2 Table**. PCR conditions were as follows: initial denaturation at 95°C for 5 min, followed by 30 cycles of 95°C for 30 s, 60°C for 30 s, 68°C for 1 min, and a final extension step of 72°C for 5 min. The amplification products were analyzed by electrophoresis (1.5% agarose gel) and cloned into a pGEM-T Easy vector (Promega) following the manufacturer’s instructions. The positive clones were identified by blue-white screening, and the sequence was confirmed by colony PCR and sequencing.

### Sequence data analysis, phylogenetic reconstruction and modeling prediction

The obtained amino acid sequence of newly identified mosquito cecropin was analyzed for its biochemical properties. The presence of a signal peptide was predicted with the SignalP 6.0 web server (https://services.healthtech.dtu.dk/services/SignalP-6.0/) using default cut-off parameters. The biochemical features of the mature peptide, including molecular weight, size, and theoretical isoelectric point, were computed using the ExPASy ProtParam Tool (http://web.expasy.org/protparam/). Grand Average of Hydropathy (GRAVY) value for peptide sequence was calculated using Gravy Calculator web server (https://www.gravy-calculator.de).

For phylogenetic analysis, maximum-likelihood reconstructions were conducted in MEGA11 [76]. Amino acid sequences of mature peptides were aligned using the MAFFT multiple alignment program (https://mafft.cbrc.jp/alignment/software). Phylogenetic inference was performed using the WAG substitution model as the best-fit model for the protein dataset, assuming uniform rates. Gaps and missing data were incorporated into data subsets as relevant phylogenetic sites, and bootstrap sampling was reiterated 1,000 times to ensure robustness.

The 3D Structure of CecD was obtained from the I-TASSER (Iterative Threading ASSEmbly Refinemen) server https://zhanggroup.org/I-TASSER/ [77], and visualized using UCSF Chimera software.

### Mosquito rearing

*Anopheles gambiae* Keele strain mosquitoes were maintained on a 10% sugar solution in laboratory culture at 27°C and 70% humidity with a 12-h light/dark cycle according to standard rearing procedures [78]. To obtain larvae for subsequent experiments, 5- to 7-day-old females were blood-fed on anesthetized mice, and freshly laid eggs were hatched in covered plastic trays (30cm x 35cm) filled with distilled water. Larval instars (approximately 150 per container) were provided with cat pellets as a nutritional source and reared under the same conditions. Water was changed every 3–4 days to avoid bacterial overgrowth.

### Spatial-temporal transcriptional profile

Larvae at each defined instar stage (L1-L4) were collected from a synchronized culture, then pooled and homogenized thoroughly in 1 mL of Trizol (Thermo, USA). Samples were stored at -80°C for subsequent total RNA extraction. For adults, gene expression of cecropin D was analyzed in either the mosquito midgut or the entire carcass. Naïve 5-day-old female mosquitoes underwent cold anesthesia, and midguts (N = 25) were dissected and combined in 1x sterile PBS. The remaining whole carcasses (N = 8) were also pooled, and the samples were thoroughly homogenized in 1 mL of Trizol and stored at -80°C. A total of three biological replicates were collected per cohort.

### Fluorescence-based reverse transcription real-time quantitative PCR (RT-qPCR)

Total RNA was purified using the TRIzol reagent (Thermo Fisher Scientific) according to the manufacturer’s specifications, and the remaining genomic DNA was removed by DNase I digestion (Thermo Fisher Scientific). First-strand cDNA was synthesized from 1 μg of total RNA using M-MLV Reverse Transcriptase (Promega) and oligo(dT)12–18 primers according to the manufacturer’s instructions. RT-qPCR amplifications were performed using the ABI StepOne Plus Real-time PCR System and SYBR Green PCR Master Mix (Thermo Fisher Scientific). Primer sequences are listed in **S2 Table**. All PCR reactions were performed in triplicate. Melting curve analysis for each primer pair was performed to ensure primer specificity. *An*. *gambiae* ribosomal S7 was used as a reference gene for RT-qPCR data normalization by the 2^−ΔΔCq^ method [79]. Statistical significance was set at *p* < 0.05 by one-way ANOVA, followed by Tukey’s multiple comparison test.

### Injection of adult mosquitoes and larvae with heat-inactivated bacteria

To assess the transcriptional response to bacterial stimulation, both L4 instars and adult mosquitoes were subjected to intra-thoracic injections of heat-killed *E*. *coli* or *S*. *aureus*. Bacterial colonies were cultured overnight in LB broth to stationary phase, washed in 1× sterile PBS, and pooled together to a final OD = 2. One-milliliter suspensions were heat-killed at 70°C for 20 min, and bacterial inactivation was confirmed on LB agar plates after overnight incubation at 37°C. For larval stimulation, L4 instars were intra-thoracically injected 24 h prior to pupation. Larvae were cold-anesthetized on a cooling Peltier block covered with moistened wipes and then microinjected with 69 nL of inactivated bacterial suspension. The injected larvae were carefully placed on a Petri dish lined with highly moistened paper towels, allowing a minimum of 10 min for the diffusion of bacteria within the larval tissues before they were transferred to clean water. Adult immune stimulation was carried out on 5-day-old females. Insects were cold-anesthetized and injected as described. Both larvae and adults were collected as four biological replicates of 10 individuals at 3-, 6-, 12-, and 24 h post-injection (hpi) and homogenized in 1 mL Trizol for downstream gene expression analyses. As an injury control, larvae and mosquitoes were injected with sterile 1× PBS.

### RNAi-based gene-silencing assays

Sense and antisense RNAs were bidirectionally synthesized from PCR-amplified gene fragments using the HiScribe T7 Quick High Yield RNA Synthesis Kit (New England Biolabs) and diluted to a final 3 μg/μL stock solution. dsRNA-mediated gene silencing was done through intra-thoracic injections into L4 instars, 48 h prior to pupation. Larvae were injected intra-thoracically with 200 ng of either dsCecD or dsGFP as a control. Four replicates of 10 injected larvae were collected at 24 h after dsRNA injection to assess gene-silencing efficiency. *An*. *gambiae* ribosomal S7 gene was employed as an internal control for qPCR data normalization. Injected larvae were monitored for survival and pupation rate over the next 6 days. Survival curves of dsCecD- and dsGFP-injected larvae were compared with a log-rank Mantel-Cox analysis using GraphPad Prism software v.9 (statistical significance, *p* < 0.05). Pupation rates were normalized daily and expressed as a percentage of total living larvae on a given day.

The bacterial loads of non-silenced and dsRNA-injected larvae were quantified by absolute quantitative PCR on purified genomic DNA. In brief, five pools of five naïve L3 and L4 instars, naïve pupae, and pupae derived from injected L4 instars were collected and washed three times with sterile PBS to remove bacterial cells attached to the larval surface. Total genomic DNA isolation was carried out with a DNeasy Blood and Tissue kit (Qiagen) according to the manufacturer’s instructions, and 10 ng of DNA was subjected to PCR amplification of 16S bacterial rDNA using universal primers (**S1 Table**). qPCR was performed as previously described. The bacterial load was estimated by logarithmic regression from a seven-point standard curve of 16S-cloned plasmid and expressed as 16S copies per ng total DNA. Statistical significance was considered *p* < 0.05 by one-way ANOVA, followed by Tukey’s multiple comparison test.

### Peptide synthesis

The mature peptide of newly characterized cecropin was synthesized by solid-phase synthesis techniques at GeneScript (Piscataway, USA). The peptide was purified to a minimum of 95% purity through high-performance liquid chromatography and delivered in the form of a lyophilized powder. Confirmation of purity was ascertained through mass spectrometry analysis. Once received, the synthetic peptide was reconstituted in ultrapure water to achieve a final stock solution of 1 mM concentration and stored at -20°C.

### Microbial culturing and antimicrobial assays

Six Gram-negative bacterial strains and four Gram-positive bacterial strains were cultured overnight to stationary phase in Luria-Bertani (LB) broth under optimal growth temperatures (28°C or 37°C) in a 200-rpm shaking incubator (**Table 1**). The yeast *Candida albicans* (ATCC SC5314) was cultivated overnight in potato dextrose broth (PDB; Sigma-Aldrich, USA) at 150 rpm in a 30°C incubator until saturation was reached. The bacterial strains and yeast suspension were stored at -80°C until used.

**Table 1:** Microbial strains used in this study.

Class	Species (strain)	Reference
Gram-negatives	*Escherichia coli* W3110	*Escherichia coli* (Migula) Castellani and Chalmers (ATCC 27325)
	*Serratia marcescens*	Dong *et al*. [81]
	*Pseudomonas aeruginosa*	Mazumdar *et al*. [82]
	*Enterobacter* sp. (*Esp_Z*)	Cirimotich *et al*. [83]
	*Chromobacterium* sp.	Ramirez *et al*. [84]
	*Elizabethkingia* sp.	Tikhe *et al*. [85]
Gram-positives	*Staphylococcus aureus*	Dong *et al*. [86]
	*Streptococcus pneumoniae*	Orihuela *et al*. [87]
	*Micrococcus luteus*	Sim *et al*. [88]
	*Bacillus subtilis*	Dong *et al*. [81]
Fungi	*Candida albicans*	ATCC SC5314
	*Beauveria bassiana*	Accoti *et al*. [89]
	*Isaria fumosorosea*	Accoti *et al*. [89]

The antibacterial activity of the synthetic peptide was assayed employing a radial diffusion method, as adapted from Kim et al. [80]. In brief, bacterial strains were cultured to exponential phase in appropriate medium at optimal growth temperature (28°C or 37°C) in a 200-rpm shaking incubator. Four milliliters of growing cultures were centrifuged, and the bacterial pellet was washed twice with 0.22 μm-filtered 1× PBS, supplemented with 5 mM glucose. Bacterial suspensions were diluted to a final OD = 0.1, and 100 μL (~ 1×10^6^ CFU) were added to 10 mL of an underlay agarose gel [0.03% (w/v) tryptic soy broth (TSB; Sigma, USA), 1% (w/v) agarose (Sigma, USA), and 0.02% (v/v) Tween 20 (Sigma, USA) in 10 mM Tris-HCl, pH 7.2] and gently poured onto 100-mm sterile polystyrene Petri dishes. After solidification, 3-mm-diameter wells were punched using sterile 3-mL syringes, and 5 μL of a 2-fold serially diluted peptide solution (100 μM to 1 μM) was added to each well. The synthetic lepidopteran cecropin A from *H*. *cecropia* (Sigma-Aldrich) was equally diluted and loaded as a positive control, and ultra-pure water was added as negative control. Plates were placed in a 28°C incubator for 3 h to allow the peptides to diffuse into the medium, and then the underlay gel was covered with 10 mL of a nutrient-rich agarose overlay gel [6% TSB, 1% agarose in 10 mM Tris -HCl]. The antimicrobial activity of the synthetic peptide was detected based on the presence of inhibitory zones around each well after 12 h of incubation at optimal bacterial growth temperature. Peptide potency was measured as the diameter of the cleared zones.

A similar radial diffusion method was also performed to assess antifungal activity against *C*. *albicans* and two filamentous entomopathogenic fungi, *Beauveria bassiana* and *Isaria fumosorosea* (**Table 1**). The frozen yeasts were thawed on ice and diluted to a final OD = 0.1, and 100 uL of suspension was mixed with 10 mL of warmed underlayer gel. Similarly, frozen spores of *B*. *bassiana* and *I*. *fumosorosea* were thawed on ice, diluted to a final concentration of 1×10^6^ spores/mL, and mixed with 10 mL of underlayer gel. Warmed potato dextrose agar (PDA: Sigma-Aldrich, USA) was used as an overlay gel to provide nutrients for the growth of both the yeasts and filamentous fungi.

### Anti-*Plasmodium* assays

To assess the anti-*Plasmodium* activity of synthetic cecropin D, 5-day-old *An*. *gambiae* females were experimentally infected with *P*. *falciparum* gametocytes, and the gametocytes were allowed to develop to the sporozoite stage. Infected mosquitoes were cold-anesthetized and subsequently dissected to provide sporozoite-infected salivary glands. The salivary glands were homogenized in sterile 1× PBS, and sporozoites were counted and adjusted to a final concentration of 6250 parasites/μL. Approximately 100,000 sporozoites were exposed to cecropin D at a range of concentrations (i.e., 200 μM, 100 μM, 50 μM and 25 μM) and incubated for 1 h at room temperature. Sterile PBS was used as control for cell viability, and 200 μM of the unrelated synthetic peptide AGAP013731, produced as an AMP candidate but shown to lack antibacterial activity, was used as a negative control. Lepidopteran cecropin A (200 μM) was also included in the experiment to allow comparison of cecropin anti-*Plasmodium* activity. After incubation, sporozoite suspensions were diluted (v:v) with the contents of a freshly prepared LIVE/DEAD Cell Imaging Kit (Thermo Fisher Scientific) as instructed by the manufacturer, and parasites were visualized under fluorescence microscopy. Parasite viability was determined by the presence of measurable green/red fluorescence, and the proportion of dead/live parasites was determined by counting fluorescent cells vs. the total sporozoite population under bright field. Following the incubations, the sporozoites were kept on ice, and cell viability was continuously monitored.

## Supporting information

S1 File Organization of cecropin coding genes of *Anopheles gambiae*. The nucleotide sequences of the cecropin genes and their respective mRNAs were aligned, and deduced amino acid sequences were determined. The nucleotide residues of mRNAs highlighted in red correspond to cecropin coding sequence (CDS). The nucleotide residues of gene sequence highlighted in italics correspond to the intronic regions. Hyphens (-) represent gaps, and the asterisk marks the stop codon. The ruler indicates the relative position of each nucleotide residue.
(DOCX)

S2 File Sequence alignment of Anopheline cecropin precursors. Conserved residues are highlighted in gray. Hyphens (-) represent gaps.
(DOCX)

S3 File Genomic location of cecropin genes within the mosquito species used in this study. (XLSX)

S4 File Antimicrobial activity of synthetic peptides as determined by radial diffusion assay. (A) Antibacterial activity of *Anopheles gambiae* cecropin D across the bacterial strains tested. (B) Antibacterial activity of positive control *Hyalophora cecropia* cecropin A peptide across the bacterial strains tested. (C) Schematic representation of peptide distribution on agar cultures for antimicrobial test.
(DOCX)

S1 Table Sequence identity matrices of precursors, mature peptides, exons and introns of *An*. *gambiae* cecropins. (XLSX)

S2 Table List of primers used in this study. (DOCX)

S1 Fig Sequence of cecropin D of *Anopheles gambiae*. mRNA sequence of *An*. *gambiae* cecropin D and deduced amino acid sequence of its precursor peptide. The nucleotide sequence represents the consensus of fully sequenced clones generated by PCR. The arrows indicate the location and orientation of specific PCR primers used for RT-qPCR (continuous line), molecular cloning (square dashed lines) and in-vitro dsRNA transcription (round dashed lines). The predicted signal peptide is highlighted in italics, with an arrowhead marking the putative signal peptidase cleavage site. The stop codon is denoted by an asterisk.
(TIF)

S2 Fig General biochemical features of *An*. *gambiae* cecropin mature peptides. Amino acid sequence of cecropin mature peptides were analyzed for their general biochemical properties. Molecular weight, theoretical isoelectric point (p*I*) and grand average of hydropathy (GRAVY) values were retrieved using Expasy ProtParam tool. Net charge at physiological pH was obtained using a public server peptide calculator.
(TIF)

S3 Fig Prediction model of cecropin D tridimensional structure. Amino acidic sequence of cecropin D was used to predict its tridimensional structure using template models available at I-TASSER server. Dark blue shows the predicted signal peptide, and light blue represents mature cecropin. Flexible hinge region is depicted in green, and the C-terminal cationic tail is shown in red. Lysine residue replacing typical tryptophan at position 2 is marked in orange. N- represents the amino-terminus. C- represents carboxy-terminus.
(TIF)

S4 Fig Molecular signatures of cecropin members. Amino acid sequences of cecropin mature peptides from diverse species of Anophelinae mosquitoes were aligned and conserved residues were identified as part of the molecular signature of each cecropin group.
(TIF)

S5 Fig Verification of gene silencing in fourth instar larvae at 24 h after dsRNA injection. Fourth instar larvae were injected with either dsCecD or dsGFP, and transcript depletion of all *An*. *gambiae* cecropin genes was assessed by qRT-PCR at 24 hpi. Transcript levels of cecropin genes of dsCecD-injected larvae were measured relative to those of the dsGFP control, and *An*. *gambiae* RpS7 was used as an internal control. Data are shown as the mean of four biological replicates ± SD. Statistical significance was determined by unpaired t-test, and significance was defined as p < 0.05. ns: not significantly different. ****, p < 0.0001.
(PNG)
