# Supplementary material for: A novel broad-spectrum antibacterial and anti-malarial Anopheles gambiae Cecropin promotes microbial clearance during pupation
Source: PLoS Pathog. 2024 Oct 23;20(10):e1012652. doi: 10.1371/journal.ppat.1012652 (PMC11554196; doi:10.1371/journal.ppat.1012652)
Supplement: S1 File — The nucleotide sequences of the cecropin genes and their respective mRNAs were aligned, and deduced amino acid sequences were determined. The nucleotide residues of mRNAs highlighted in red correspond to cecropin coding sequence (CDS). The nucleotide residues of gene sequence highlighted in italics correspond to the intronic regions. Hyphens (-) represent gaps, and the asterisk marks the stop codon. The ruler indicates the relative position of each nucleotide residue. (DOCX) [file ppat.1012652.s001.docx]

**S1 File. Organization of cecropin coding genes of *Anopheles gambiae*.** The nucleotide sequences of the cecropin genes and their respective mRNAs were aligned, and deduced amino acid sequences were determined. The nucleotide residues of mRNAs highlighted in red correspond to cecropin coding sequence (CDS). The nucleotide residues of gene sequence highlighted in italics correspond to the intronic regions. Hyphens (-) represent gaps, and the asterisk marks the stop codon. The ruler indicates the relative position of each nucleotide residue.

**Cecropin A [AGAP000693]**

**10 20 30 40 50 60 70 80 90 100**

**....|....|....|....|....|....|....|....|....|....|....|....|....|....|....|....|....|....|....|....|**

**CecA_gene** -CGTTCCTGCAGCAAACATCTTATCAACCCAGAGACCAACCAACCACCAAACAACCCAACAACAATGAACTTCTCCAAGATCTTCATCTTTGTCGTGCTG

**CecA_mRNA** GCGTTCCTGCAGCAAACATCTTATCAACCCAGAGACCAACCAACCACCAAACAACCCAACAACAATGAACTTCTCCAAGATCTTCATCTTTGTCGTGCTG

M N F S K I F I F V V L

**110 120 130 140 150 160 170 180 190 200**

**....|....|....|....|....|....|....|....|....|....|....|....|....|....|....|....|....|....|....|....|**

**CecA_gene** GCAGTGCTGTTGCTCTGCAGTCAGACGGAAGCGGGACGGCTGAAGAAGCTGGGAAAGAAAATT*GTAAGTACTAGATGGGTTGCACCGTGCGGAGGAGCTT*

**CecA_mRNA** GCAGTGCTGTTGCTCTGCAGTCAGACGGAAGCGGGACGGCTGAAGAAGCTGGGAAAGAAAATT-------------------------------------

A V L L L C S Q T E A G R L K K L G K K I

**210 220 230 240 250 260 270 280 290 300**

**....|....|....|....|....|....|....|....|....|....|....|....|....|....|....|....|....|....|....|....|**

**CecA_gene** *CTACAACAAGGCTCACTGTTGCTGATGTTTGTTAAAACTTCATTGTCCACAG*GAGGGAGCCGGCAAGCGAGTGTTCAAGGCAGCAGAGAAGGCCCTACCG

**CecA_mRNA** ----------------------------------------------------GAGGGAGCCGGCAAGCGAGTGTTCAAGGCAGCAGAGAAGGCCCTACCG

E G A G K R V F K A A E K A L P

**310 320 330 340 350 360 370 380 390 400**

**....|....|....|....|....|....|....|....|....|....|....|....|....|....|....|....|....|....|....|....|**

**CecA_gene** GTGGTGGCAGGCGTTAAGGCGCTCGGTTAGAGCGTCGGCAGGAGGATGGTTCTATTCGTGCTTAAGACGACGGCTCTGCTAACATGATATGGCAAAGAGA

**CecA_mRNA** GTGGTGGCAGGCGTTAAGGCGCTCGGTTAGAGCGTCGGCAGGAGGATGGTTCTATTCGTGCTTAAGACGACGGCTCTGCTAACATGATATGGCAAAGAGA

V V A G V K A L G *

**410 420 430 440 450 460 470 480 490 500**

**....|....|....|....|....|....|....|....|....|....|....|....|....|....|....|....|....|....|....|....|**

**CecA_gene** GAGAGAGAGAGAGAGAGAGAGAGAGAGAGAGAGAGAGAGAGAGAGAGAGAGAGAGAGAGAGAGAGAGAGAGAGAGAGGGAGAGAGATGAATCAGCTTTAA

**CecA_mRNA** GAGAGAGAGAGAGAGAGAGAGAGAGAGAGAGAGAGAGAGAGAGAGAGAGAGAGAGAGAGAGAGAGAGAGAGAGAGAGGGAGAGAGATGAATCAGCTTTAA

**510 520 530 540 550 560 570 580 590 600**

**....|....|....|....|....|....|....|....|....|....|....|....|....|....|....|....|....|....|....|....|**

**CecA_gene** CAGATCTGTGGGCAGGGAGGGGAAAAGTGATAAAATTATAGCATGTAGTAAACAGATCTTGACTAGTTCGGCAAATAAATTTCACTTCTTATCTAACCGC

**CecA_mRNA** CAGATCTGTGGGCAGGGAGGGGAAAAGTGATAAAATTATAGCATGTAGTAAACAGATCTTGACTAGTTCGGCAAATAAATTTCACTTCTTATCTAACCGC

**Cecropin B [AGAP000694]**

**10 20 30 40 50 60 70 80 90 100**

**....|....|....|....|....|....|....|....|....|....|....|....|....|....|....|....|....|....|....|....|**

**CecB_gene** CTGAGATCTCTTCCCGTGTGGAGCAAATCGATTCCCAGTGCATCTGTGTCCAAAACCAAGAGCTAACAATGAACTTCACCAAGCTGTTCATCCTGGTGGC

**CecB_mRNA** CTGAGATCTCTTCCCGTGTGGAGCAAATCGATTCCCAGTGCATCTGTGTCCAAAACCAAGAGCTAACAATGAACTTCACCAAGCTGTTCATCCTGGTGGC

M N F T K L F I L V A

**110 120 130 140 150 160 170 180 190 200**

**....|....|....|....|....|....|....|....|....|....|....|....|....|....|....|....|....|....|....|....|**

**CecB_gene** GATTGCGGTCCTGGTGGTCGTTGGCGTACAGCCAGTCGATGGTGCGCCGCGGTGGAAGTTCGGCAAGCGGTTG*GTAAGAATAACATTTGTTTTTTTTTTC*

**CecB_mRNA** GATTGCGGTCCTGGTGGTCGTTGGCGTACAGCCAGTCGATGGTGCGCCGCGGTGGAAGTTCGGCAAGCGGTTG---------------------------

I A V L V V V G V Q P V D G A P R W K F G K R L

**210 220 230 240 250 260 270 280 290 300**

**....|....|....|....|....|....|....|....|....|....|....|....|....|....|....|....|....|....|....|....|**

**CecB_gene** *TTCTTCTTCTTGGCCATACACCAGAAATAGTGATTCTAACCATTTTTTTCTATTATTCTGATTCACAG*GAGAAGCTCGGACGCAATGTGTTTCGGGCGGC

**CecB_mRNA** --------------------------------------------------------------------GAGAAGCTCGGACGCAATGTGTTTCGGGCGGC

E K L G R N V F R A A

**310 320 330 340 350 360 370 380 390 400**

**....|....|....|....|....|....|....|....|....|....|....|....|....|....|....|....|....|....|....|....|**

**CecB_gene** TAAGAAAGCGCTGCCCGTCATTGCCGGCTACAAAGCTCTTGGATAGGATGTGGCTGACTGATCCCGACCCGACCGCTCAACTAGTGATACAGAACAGCAA

**CecB_mRNA** TAAGAAAGCGCTGCCCGTCATTGCCGGCTACAAAGCTCTTGGATAGGATGTGGCTGACTGATCCCGACCCGACCGCTCAACTAGTGATACAGAACAGCAA

K K A L P V I A G Y K A L G *

**410 420 430 440 450 460 470 480 490 500**

**....|....|....|....|....|....|....|....|....|....|....|....|....|....|....|....|....|....|....|....|**

**CecB_gene** ACGTAGCGCAGTGACACCCTACTCCTAATGGTATCCATTATCAAAATGTTTCACTGATCAACCCGCAGTGGTATTAATCGTAAGACTGAAAGAGGTCACC

**CecB_mRNA** ACGTAGCGCAGTGACACCCTACTCCTAATGGTATCCATTATCAAAATGTTTCACTGATCAACCCGCAGTGGTATTAATCGTAAGACTGAAAGAGGTCACC

**510 520 530 540 550 560**

**....|....|....|....|....|....|....|....|....|....|....|....|....|...**

**CecB_gene** GCCTCCGCCGGTTCGGTGGAGTGATATTGGCAAACAGCTGCATCGACAATGTCCACGTTTTACTGTGT

**CecB_mRNA** GCCTCCGCCGGTTCGGTGGAGTGATATTGGCAAACAGCTGCATCGACAATGTCCACGTTTTACTGTGT

**Cecropin C [AGAP000692]**

**10 20 30 40 50 60 70 80 90 100**

**....|....|....|....|....|....|....|....|....|....|....|....|....|....|....|....|....|....|....|....|**

**CecC_gene** GCGCCCGGTTGACTGAGAGCGATTCACTAATTGTTCCGGCACCTGCTTCGCGAGTGTAAAACCAACCGAAAGTTCGAACAGCAGCACCATGAACTTCAAG

**CecC_mRNA** GCGCCCGGTTGACTGAGAGCGATTCACTAATTGTTCCGGCACCTGCTTCGCGAGTGTAAAACCAACCGAAAGTTCGAACAGCAGCACCATGAACTTCAAG

M N F K

**110 120 130 140 150 160 170 180 190 200**

**....|....|....|....|....|....|....|....|....|....|....|....|....|....|....|....|....|....|....|....|**

**CecC_gene** CTGATCTTTCTCGTCGCGCTGGTACTGATGGCTGCGTTCCTGGGCCAAACCGAGGGTCGTCGGTTCAAAAAGTTCCTGAAGAAAGTG*GTGTGTACTGGGA*

**CecC_mRNA** CTGATCTTTCTCGTCGCGCTGGTACTGATGGCTGCGTTCCTGGGCCAAACCGAGGGTCGTCGGTTCAAAAAGTTCCTGAAGAAAGTG-------------

L I F L V A L V L M A A F L G Q T E G R R F K K F L K K V

**210 220 230 240 250 260 270 280 290 300**

**....|....|....|....|....|....|....|....|....|....|....|....|....|....|....|....|....|....|....|....|**

**CecC_gene** *TCGGCGGCAGTGTTGGCGATTTGCTGTCTACATCATCACACCGATGACTAATGCATTATACATCTCCATTGCAG*GAAGGCGCAGGCCGACGAGTAGCCAA

**CecC_mRNA** --------------------------------------------------------------------------GAAGGCGCAGGCCGACGAGTAGCCAA

E G A G R R V A N

**310 320 330 340 350 360 370 380 390 400**

**....|....|....|....|....|....|....|....|....|....|....|....|....|....|....|....|....|....|....|....|**

**CecC_gene** TGCAGCCCAGAAGGGTTTGCCGCTGGCGGCGGGCGTAAAGGGTCTCGTTGGATAATGGGTCCCCCTAGCCAGCTTCTTGTTGCGTTCCCTCCCGAAGGTG

**CecC_mRNA** TGCAGCCCAGAAGGGTTTGCCGCTGGCGGCGGGCGTAAAGGGTCTCGTTGGATAATGGGTCCCCCTAGCCAGCTTCTTGTTGCGTTCCCTCCCGAAGGTG

A A Q K G L P L A A G V K G L V G *

**410 420 430 440 450 460 470 480 490 500**

**....|....|....|....|....|....|....|....|....|....|....|....|....|....|....|....|....|....|....|....|**

**CecC_gene** GGGAGTTCTCTCCACCCGCTCCAAACCGATCACGATCCCTCTGCAATCAATTCCGTCACCCGACACCGACGTCAACGAACTGGCGCGGTACAGTGTTTAC

**CecC_mRNA** GGGAGTTCTCTCCACCCGCTCCAAACCGATCACGATCCCTCTGCAATCAATTCCGTCACCCGACACCGACGTCAACGAACTGGCGCGGTACAGTGTTTAC

**510 520 530 540 550 560 570 580 590 600**

**....|....|....|....|....|....|....|....|....|....|....|....|....|....|....|....|....|....|....|....|**

**CecC_gene** TACAACGCCTTAATCAACGCCACCGTGCAAGGCACAACCGTTCCAATAAAGTGCCTGTCTCGGGCCAACTTCCTTAATACGTGAGCAGTTCAAACCTCGG

**CecC_mRNA** TACAACGCCTTAATCAACGCCACCGTGCAAGGCACAACCGTTCCAATAAAGTGCCTGTCTCGGGCCAACTTCCTTAATACGTGAGCAGTTCAAACCTCGG

**....|**

**CecC_gene** CGGCC

**CecC_mRNA** CGGCC

**Cecropin D [AGAP006722]**

**10 20 30 40 50 60 70 80 90 100**

**....|....|....|....|....|....|....|....|....|....|....|....|....|....|....|....|....|....|....|....|**

**CecD_gene** ACAGTGATTCCATCTCGAACGGGAAAGTGACAGCTGCACACTCGGGGTTTAAGCTGAACTGAAAGGAAGCTGCAAAACAACAAGCATCGTTTGCCAAAGC

**CecD_mRNA** ------------------------------------TACACTCGGGGTTTAAGCTGAACTGAAAGGAAGCTGCAAAACAACAAGCATCGTTTGCCAAAGC

**110 120 130 140 150 160 170 180 190 200**

**....|....|....|....|....|....|....|....|....|....|....|....|....|....|....|....|....|....|....|....|**

**CecD_gene** CTCAAAATGAACGTATCGAAGCTGTTTGTTATCGTCCTGCTGGCCACGCTGCTACTGTTCGGTGGTCAAGCCGAGGCGGGCCATCTGAAAAAGTTTGGCA

**CecD_mRNA** CTCAAAATGAACGTATCGAAGCTGTTTGTTATCGTCCTGCTGGCCACGCTGCTACTGTTCGGTGGTCAAGCCGAGGCGGGCCATCTGAAAAAGTTTGGCA

M N V S K L F V I V L L A T L L L F G G Q A E A G H L K K F G

**210 220 230 240 250 260 270 280 290 300**

**....|....|....|....|....|....|....|....|....|....|....|....|....|....|....|....|....|....|....|....|**

**CecD_gene** AAAAATTG*GTAAGCAACTAGCGATGGTAGCGAGCGGCCGGGCATTATCCCTTTCAGCTAAGGGCTGTTGATTTGCTCACTGTATGATTGTTTCATTTTAT*

**CecD_mRNA** AAAAATTG--------------------------------------------------------------------------------------------

K K L

**310 320 330 340 350 360 370 380 390 400**

**....|....|....|....|....|....|....|....|....|....|....|....|....|....|....|....|....|....|....|....|**

**CecD_gene** *CTCTCCCTATTTCTTTCCACTCTCGTTCACCGCGGGCGCAG*GAAAAGGTTGGCAAAAATGTGTTCCACGCAGTGGAAAAGGTAGTGCCCGTACTGCAGGG

**CecD_mRNA** -----------------------------------------GAAAAGGTTGGCAAAAATGTGTTCCACGCAGTGGAAAAGGTAGTGCCCGTACTGCAGGG

E K V G K N V F H A V E K V V P V L Q G

**410 420 430 440 450 460 470 480 490 500**

**....|....|....|....|....|....|....|....|....|....|....|....|....|....|....|....|....|....|....|....|**

**CecD_gene** TATACAGGATTTGCGTGACAAGAAGAACGGTCAAAGGGGTTAGCAAGGAACAGTCCGGTGGCTCGGAGGCTTGTTCAACAAGCTCGCAAATGGAGAGACA

**CecD_mRNA** TATACAGGATTTGCGTGACAAGAAGAACGGTCAAAGGGGTTAGCAAGGAACAGTCCGGTGGCTCGGAGGCTTGTTCAACAAGCTCGCAAATGGAGAGACA

I Q D L R D K K N G Q R G *

**510 520 530 540 550 560 570 580 590**

**....|....|....|....|....|....|....|....|....|....|....|....|....|....|....|....|....|....|....**

**CecD_gene** GTTTGAAGTCCCTGGCTGGACTGTTTTTGAGCCTGCAACCGTCACAAACATCAAAGGACTGTCTTTGCAGCCTGCGCAGCGAGATGAAGATTAT

**CecD_mRNA** GTTTGAAGTCCCTGGCTGGACTGTTTTTGAGCCTGCAACCGTCACAAACATCAAAGGACTGTCTTTGCAGCCTGCGCAGCGAGATGAAGATTAA
