# Supplementary material for: A novel broad-spectrum antibacterial and anti-malarial Anopheles gambiae Cecropin promotes microbial clearance during pupation
Source: PLoS Pathog. 2024 Oct 23;20(10):e1012652. doi: 10.1371/journal.ppat.1012652 (PMC11554196; doi:10.1371/journal.ppat.1012652)
Supplement: S2 File — Conserved residues are highlighted in gray. Hyphens (-) represent gaps. (DOCX) [file ppat.1012652.s002.docx]

**S2 File. Sequence alignment of Anopheline cecropin precursors.** Conserved residues are highlighted in gray. Hyphens (-) represent gaps.

**CECROPIN A**

**AGAP000693_[Anopheles gambiae]** MNFSKIFIF-VVLAVLLLC-SQTEAGRLKKLGKKIEGAGKRVFKAAEKALPVVAGVKALG-

**XP_050099629.1_[Anopheles aquasalis]** MNFSKVLFV-VVVAALLLI-GQADAGFLKKLGKKIEGAGKRVFNAAEKALPVAVSVKALGK

**XP_050087634.1_[Anopheles aquasalis]** MNFSKLVLFALLIATLLLV-GQIEAGRLKKLGKKIEGAGKRVFKAAEKTLPVAVGVKALGK

**XP_058127228.1_[Anopheles coustani]** MNFNKIFVF-VLLAALVLV-GQTEAGWLKKLGKKIEGAGKRVFKATEKALPVIAGVKALG-

**XP_058127091.1_[Anopheles coustani]** MNFNKIFVF-VLLAALVLL-GQTEAGRLKKLGKKIEGAGKRVFKAAEKALPVVAGAKALG-

**XP_058175824.1_[Anopheles ziemanni]** MNFNKIFVF-VLLAALVLV-GQTEAGWLKKLGKKIEGAGKRVFKATEKALPVIAGVKALG-

**XP_058175502.1_[Anopheles ziemanni]** MNFNKIFVF-VLLAALVLL-GQTEAGRLKKLGKKIEGAGKRVFKAAEKALPVVAGAKALG-

**XP_058063586.1_[Anopheles bellator]** MNFSKIFLF-VVLATLLLAVGQTEAGWLKKLGKKVEKAGQRVFNAAEKALPVATGVKALGK

**XP_052867605.1_[Anopheles cruzii]** MNFSKIFLF-VVLATLLLAVGQTEAGWLKKLGKKVEKAGQRVFNAAEKALPVATGIKALGK

**XP_049538583.1_[Anopheles darlingi]** MNFNKILVLAVVLAALLLV-GQTDAGFLKKLGKKIEGAGKRVFNAAEKALPVAVGVKALGK

**XP_052901011.1_[Anopheles moucheti]** MNFTKIFIF-VVLAALLLC-GQTEAGWLKKLGKKVEGAGKRVFNAAEKALPVAAGVKALG-

**XP_035773329.1_[Anopheles albimanus]** MNFNKVLVL-VVVAALLLV-GQVDAGFLKKLGKKIEGAGKRVFNAAEKALPVAVGVKALGK

**XP_040173531.1_[Anopheles arabiensis]** MNFSKIFIF-VVLAVLLLC-SQTEAGRLKKLGKKIEGAGKRVFKAAEKALPVVAGVKALG-

**XP_049282029.1_[Anopheles funestus]** MNFNKIFIF-VVLAVLLLC-SQTEAGRLKKLGKKIEGAGKRVFKAAEKALPVVAGVKALG-

**XP_040229870.1_[Anopheles coluzzi]** MNFSKIFIF-VVLAVLLLC-SQTEAGRLKKLGKKIEGAGKRVFKAAEKALPVVAGVKALG-

**XP_035903635.1_[Anopheles stephensi]** MNFNKIFVF-VVLAVLLLC-SQTEAGRLKKLGKKIEGVGKRVFKAAEKALPVVAGVKALG-

**XP_041774550.1_[Anopheles merus]** MNFSKIFIF-VVLAVLLLC-SQTEAGRLKKLGKKIEGVGKRVFKAAEKALPVVAGVKALG-

**XP_053664686.1_[Anopheles marshallii]** MNFTKIFIF-VVLAALLLC-GQTEAGRLKKLGKKIEGAGKRVFKAAEKALPVAAGVKALG-

**XP_050073979.1_[Anopheles maculipalpis]** MNFNKIFLF-VVLAVLLLC-SQTEAGRLKKLGKKIEGAGKRVFKAAEKALPVAVGVKALG-

**ADIR015737_[Anopheles dirus]**  MNFGKIFIF-VVLAALLLF-GQAEAGGLKKLGKKLEGAGKRVFKAAEKALPVAAGVKALG-

**ADIR015738_[Anopheles dirus]**  MNLGKIFIF-VVLAALLFL-GQAEAGRLRNFGKKLERGGKRVFKAAAKVLPVVAGFKALG-

**AFAF021636_[Anopheles farauti]** MNFGKIFVF-VVLAALLLL-GQTEAGKLKKLGKKLEGAGKRVFKAAEKALPVIAGVKALG-

**ACHR015125_[Anopheles christyi]** MNFTKIFIF-VVLAVLLLC-GQTEAGRLKKLGKKIEGAGKRVFKAAEKALPVVAGVKALG-

**AMEC023154_[Anopheles melas]**  MNFSKIFFI-VVLAVLLLC-SQTEAGRLKKLGKKIEGAGKRVFKAAEKALPVVAGVKALG-

**AQUA015912_[Anopheles quadriannulatus]** MNFSKIFIF-VVLAVLLLC-SQTEAGRLKKLGKKIEGAGKRVFKAAEKALPVVAGVKALG-

**AMIN015909_[Anopheles minimus]** MNFTKIFIF-VVLAALLLC-GQTEAGRLKKLGKKIEGAGKRVFKAAEKALPVVAGVKALG-

**ACUA029182_[Anopheles culicifacies]**  MNFTKIFIF-VVLAALLLC-GQTEAGRLKKLGKKIEGAGKRVFKAAEKALPVVAGVKALG-

**AEPI014449_[Anopheles epiroticus]** MNFTKIFVF-LVLAVLLFA-GHTEAGRLKKLGKKIEGAGKRVFKAAEKALPVIAGVKALG-

**ABU80042.1_[Anopheles bwambae]** MNFSKIFIF-VVLAVLLLC-SQTEAGRLKKLGKKIEGVGKRVFKAAEKALPVVAGVKALG-

**AATE021908_[Anopheles atroparvus]** MNFNKLFVF-VLLAALLLL-GQTEAGRLKKLGKKIEGVGKRVFKAAEKALPVVAGVKALGR

**KFB39637.1_[Anopheles sinensis]** MNFNKIFVF-VLLAALVLL-GQTEAGRLKKLGKKIEGVGKRVFKAAEKALPVVAGVKALG-

**Cecropin B**

**AGAP000694_[Anopheles gambiae]** MNFTKLFILVAIAVLVVVGVQPVDGAPR-WKFGKRLEKLGRNVFRAAKKALPVIAGYKA-LG

**XP_053678472.1_[Anopheles nili]** MNFKLIFLVSMLLMAVFLGQS--EAAPF-RKFVRRLRGAGRRVGNVVRKVAPVVAGAKAVIG

**XP_053678471.1_[Anopheles nili]** MNFTKLFILVAIAVLVIAGVHQADGAPR-WKFGKRLEKLGRNVFRAAKKALPVIAGYKA-LG

**XP_049538569.1_[Anopheles darlingi]** MNFTKLFILVAIAVLVIAGIQPADAAPR-WKFGKRLERLGRNVFKAAKKALPVIAGYKA-LG

**XP_035773307.1_[Anopheles albimanus]** MNFTKLFIMVAIAVLLIAGIQPVEAAPR-WKFGKRLEKLGRNVFKAAKKALPVIAGYKA-LG

**XP_050087620.1_[Anopheles aquasalis]** MNFTKLFIVVAIAVLVIAGIQPADAAPR-WKFGKRLEKLGRNVFKAAKKALPVIAGYKA-LG

**XP_041774540.1_[Anopheles merus]** MNFTKLFILVAIAVLVVVGVQPVDGAPR-WKFGKRLEKLGRNVFRAAKKALPVVAGYKA-LG

**XP_035903610.1_[Anopheles stephensi]** MNFTKLFILVAIAVLVIVGVQPVDGAPR-WKFGKRLEKLGRNVFRAAKKALPVVAGYKA-LG

**XP_052901010.1_[Anopheles moucheti]** MNFTKLFILVAIAVLVIVGVQPADGAPR-WKFGKRLEKLGRNVFRAAKKALPVIAGYKA-LG

**XP_049282040.1_[Anopheles funestus]** MNFTKLFILVAIAVLVIVGVQPADGAPR-WKFGKRLEKLGRNVFRAAKKALPVIAGYKA-LG

**XP_040229856.1_[Anopheles coluzzi]** MNFTKLFILVAIAVLVVVGVQPVDGAPR-WKFGKRLEKLGRNVFRAAKKALPVIAGYKA-LG

**XP_040173530.1_[Anopheles arabiensis]** MNFTKLFILVAIAVLVVVGVQPVDGAPR-WKFGKRLEKLGRNVFRAAKKALPVIAGYKA-LG

**XP_053659077.1_[Anopheles marshallii]** MNFTKLFILVAIAVLVIVGVQPADGAPR-WKFGKRLEKLGRNVFRAAKKALPVIAGYKA-LG

**XP_050073956.1_[Anopheles maculatus** MNFTKLFILVAIAVLVVVGVQPVDGAPR-WKFGKRLERLGRNVFRAAKKALPVIAGYKA-LG

**XP_058063719.1_[Anopheles bellator]** MNFTKLFILVAIAVLVVVGVQPADGAPR-WKFGKRLEKLGRNVFKAAQKALPVIAGYKA-LG

**XP_052862118.1_[Anopheles cruzi]** MNFTKLFILVAIAVLVVVGVQPADGAPR-WKFGKRLEKLGRNVFKAAQKALPVIAGYKA-LG

**AFAF010844_[Anopheles farauti]** MNFTKIFLLVAIAVLVVVGVQPVDGAPR-WKFGKRLEKLGRNVFRAAQKALPVIAGYKA-LG

**AMIN005847_[Anopheles minimus]** MNFAKLFILVAIAVLVIVGVQPVEGAPR-WKFGKRLEKLGRNVFRAAQKALPVIAGYKA-LG

**ACUA029183_[Anopheles culicifacies]** MNFAKLFILVAIAVLVIVGVQPVEGAPR-WKFGKRLEKLGRNVFRAAQKALPVIAGYKA-LG

**ADIR000944_[Anopheles dirus]**  MNFTKLFLLVAIAVLVVVGVQPVEGAPR-WKFGKRLERLGRNVFRAAKKALPVIQGYKA-LG

**AATE021906_[Anopheles atroparvus]** MNFRRLFVLVVLVALVMVGVQPADGAPRRWRFGKRLEKLGRNIFRAAQKALPVIQGYKA-LG

**ACHR009161_[Anopheles christyi]** MNFGKLFILVAIAVLVVVSVQPVDGAPR-WKFGKRLEKLGRNVFRAAKKALPVVAGYKA-LG

**AMEC023330_[Anopheles melas]**  MNFTKLFILVAIAVLVVVGVQPADGAPR-WKFGKRLEKLGRNVFRAAKKALPVVAGYKA-LG

**AEPI011006_[Anopheles epiroticus]** MNFAKLFILVAIAVLVVVGVQPVDGAPR-WKFGKRLEKLGRNVFRAAKKALPVVAGYKA-LG

**AQUA005774_[Anopheles quadriannulatus]** MNFTKLFILVAIAVLVVVGVQPVDGAPR-WKFGKRLEKLGRNVFRAAKKALPVVAGYKA-LG

**AMAM024566_[Anopheles maculatus]** MNFTKLFILVAIAVLVVVGVQPADGAPR-WKFGKRLEKLGRNVFRAAKKALPVIAGYKA-LG

**Cecropin C**

**AGAP000692_[Anopheles gambiae]** MNFKLIFLVALVLMAAFLGQT-EGRRFKKFLKKVEGAGRRVANAAQKGLPLAAGVKGLVG

**AMIN015908_[Anopheles minimus]** MNFKLIFLVALVLMAVCLGQS-EGRRFKKFLKKVQGAGRRVANAAQKGLPLALGVKGVLG

**AMAM024572_[Anopheles maculatus]** MNFKLLFLVALVLMAVCLGQS-EGRRFKKFLKKVEGAGRRVANAAQKGLPLALGVKGVLG

**ACUA029184_[Anopheles culicifacies]** MNFKLIFLVALVLMAVCLGQS-EGRRFKKFLKKVEGAGRRVANAAQKGLPLALGVKGVLG

**XP_035903623.1_[Anopheles stephensi]** MNFKLLFLVALVLMAVCLGQS-EGRRFKKFLKKVEGAGRRVAGAAQKGLPLALGVKGVLG

**XP_040172706.1_[Anopheles arabiensis]** MNFKLIFLVALVLMAAFLGQT-EGRRFKKFLKKVEGAGRRVANAAQKGLPLAAGVKGLVG

**XP_040221939.1_[Anopheles coluzzi]** MNFKLIFLVALVLMAAFLGQT-EGRRFKKFLKKVEGAGRRVANAAQKGLPLAAGVKGLVG

**XP_041768900.1_[Anopheles merus]** MNFKLIFLVALVLMAAFLGQT-EGRRFKKFLKKVEGAGRRVANAAQKGLPLAAGVKGLVG

**AQUA015913_[Anopheles quadriannulatus]** MNFKLIFLVALVLMAAFLGQT-EGRRFKKFLKKVEGAGRRVANAAQKGLPLAAGVKGLVG

**ACHR009160_[Anopheles christyi]** MNFKLIFLVALVLMAAFLGQT-EGRRFKKFLKKVEGAGRRVANAAQKGLPLAAGVKGLAG

**AEPI011007_[Anopheles epiroticus]** MNFKLIFLVALVLMAAFLGQTVEGRRFKKFLKKVEGAGRRVANAAQKGLPLALGVKGLAG

**XP_058063871.1_[Anopheles bellator]** MNLKLFLIVSLLLVGVFLG-QTEGRRFKKFLKKVEGAGRRITNAAQKGLPVAAGVKGLVG

**XP_049538560.1_[Anopheles darlingi]** MQLKVILLVALVLMAALFGGETEARRFRKFLKKVEGAGRRITNAAQKGLPVVAGVKGIIG

**XP_035773319.1_[Anopheles albimanus]** MQLKVILLVALVLMATLLGGQTEARRFKKFLKKVEGAGRRITNAAHKGLPVVAGVKGIIG

**XP_052862883.1_[Anopheles cruzi]** MNLKLFLIVSLLLVGVFLG-QTEGRRFKKFLKKVEGAGRRITNAAQKGLPVVAGAQGLIG

**AMEC023138_[Anopheles melas]**  MNFKLIFLVALVLMAAFLGQT-EGRRFKKFLKK-EGAGRRVANAAQKGLPLAAGVKGLVG

**XP_050073968.1_[Anopheles maculatus]** MNFKLLFLVALVLMAVCLGQT-EGRRFKKFLKKVGGAGRRVANAAQKGLPLALGVRGALG

**ADIR015736_[Anopheles dirus]**  MNFKLIFLVTLLLAAVFLGQT-EGRRFKKFLKKVEGAGRRVTNAAQKGLPVVAGASAVLG

**AFAF011386-[Anopheles farauti]** MNFKLIFLVSLLLAAIFLDPT-EGRRFKKFLKKVEGVGKRVRNAAQKGLPLAAGAQAVLG

**XP_053664034.1_[Anopheles marshallii]** MNFKLIFLVALVLMAVCLGES-EGRPFKKFLRKLRGAGRRVANAAQKAAPLALGVRGVLG

**XP_058128235.1_[Anopheles coustani]** MNFKLILIVSLMLMALFFG---EAECLRKRLRRLGRAGRRIANAAQKIAPVVGALRALG-

**KFB41929.1_[Anopheles sinensis]** MNFKLILIVSLVLMALFFG---EAECFRKRLRRLGRAGRRIAKAVQKVAPVVGAVRAIG-

**AATE021907_[Anopheles atroparvus]** MNFKLILVVSLMLAALFFG---EAECFRRTLRRLGRFGRRVGKVAQKVAPVVGGVRAVVG

**Cecropin D**

**AGAP006722_[Anopheles gambiae]** MNVSKLFVIVLLATLLLFGGQAEAGHLKKFGKKLEKVGKNVFHAVEKVVPV-LQGIQDLRDKKNGQRG-

**XP_035793397.1_[Anopheles albimanus]** MNLTKLFVVLMLVAAVLFGGQAEAGHLKKFGKKLEKVGQRVFKATEKVVPV-LLGIKQLGQKKD-----

**XP_035793395.1_[Anopheles albimanus]** MNLTKLFVVLTLVAAVLFSGQAEAGHVEKFVKKPGKVEKQKFKVKESIKTQNIKRVIKFNGKEHRATQ-

**XP_050092943.1_[Anopheles aquasalis]** MNLTKLFVVVLLAMAVLFGGQVEAGRLKNFGKKLEKVGQRVFKATEKVVPV-LLGIKQLGKKDDGH---

**XP_049542450.1_[Anopheles darlingi]** MNLTKLFVVLLLVTAVLFGGQVEAGHLRKFGKKLEKVGQRVFKATEKVVPV-LLGIKQLGRKEDGQ---

**XP_040160406.1_[Anopheles arabiensis]** MNVSKLFVIVLLATLLLLDGQAEAGHLKKFGKKLEKVGKNVFHAVEKVVPV-LQGIQDLREKKNGQRG-

**XP_040241448.2_[Anopheles coluzzi]** MNVSKLFVIVLLATLLLFGGQAEAGHLKKFGKKLEKVGKNVFHAVEKVVPV-LQGIQDLRDKKNGQRG-

**XP_041774688.1_[Anopheles merus]** MNVSKLLVIVLLATLLLFGGQAEAGHLKKFGKKLEKVGKNVFHAVEKVVPV-LQGIQDLRDKKNGQRG-

**XP_053666749.1_[Anopheles marshallii]** MNLSKLFIVVLLGMLLLFAGQTEAGHLKKFGKKLEKIGKNVFHAVEKVVPV-LQGVQDLRDGKKG----

**XP_035906979.1_[Anopheles stephensi]** MNLSKLFIVLLLGTLLLFGGQTEAGHLKKFGKKLEKIGKNVFHAVEKVVPV-LAGVQDLREKQKG----

**AFAF021902_[Anopheles farauti]** MNLSKLFVVVLLATLLLFGTQTEAGHLKKFGKKLEKIGKNVFHAVEKVVPV-LQGIQDLKKGQNGQSG-

**ADIR003663_[Anopheles dirus]**  MNLSKLFIIVLLATLLLFGGQTEAGHLKKFGKKLEKMGQNVFHAVQKVVPV-LQEIQDLRNGEKKPPGQ

**AEPI015479_[Anopheles epiroticus]** MNLSKLLVVMLLATLLLFGGHTEAGVLKKLGKKLEKVGKNVLRAAERVVPL-LLSIQDLRDKEKRLRG-

**AATE007875_[Anopheles atroparvus]** MKLTKLFFIVLLATVLLFGGQAEAGGLKHLGKHLEKMGQNVLHATEKVVHV-LKEVKDLTKKDTNDS--

**AMEC023366_[Anopheles melas]**  MNVSKLFVIVLLATLLLFGGQAEAGHLKKFGKKLEKVGKNVFHAVEKVVPV-LQGIQDLRDKKNGQRG-

**AQUA003575_[Anopheles quadriannulatus]** MNVSKLFVIVLLATLLLFGGQAEAGHLKKFGKKLEKVGKNVFHAVEKVVPV-LQGIQDLRDKKNGQRG-

**ACUA029192_[Anopheles culicifacies]** MNLSKLFIVILLGTLLLFGGQTEAGHLKKFGKKLEKIGKNVFHAVEKVVPV-LQGVQDIRDGKKG----

**AMIN010113_[Anopheles minimus]** MNLSKLFIVVLLGTLLLFGGQTEAGHLKKFGKKLEKIGKNVFHAVEKVVPV-LQGVQDIRDNKNGQTG-

**AMAM024573_[Anopheles maculatus]** MNLSKLLIVVLLGALLLFAGQTEAGHLKKFGKKLEKIGKNVFHTVEKVVPV-LAGVQDLREGKKG----
