# Supplementary material for: A novel broad-spectrum antibacterial and anti-malarial Anopheles gambiae Cecropin promotes microbial clearance during pupation
Source: PLoS Pathog. 2024 Oct 23;20(10):e1012652. doi: 10.1371/journal.ppat.1012652 (PMC11554196; doi:10.1371/journal.ppat.1012652)

**S4 File.** **Antimicrobial activity of synthetic peptides as determined by radial diffusion assay.** (A) Antibacterial activity of *Anopheles gambiae* cecropin D across the bacterial strains tested. (B) Antibacterial activity of positive control *Hyalophora cecropia* cecropin A peptide across the bacterial strains tested. (C) Schematic representation of peptide distribution on agar cultures for antimicrobial test.


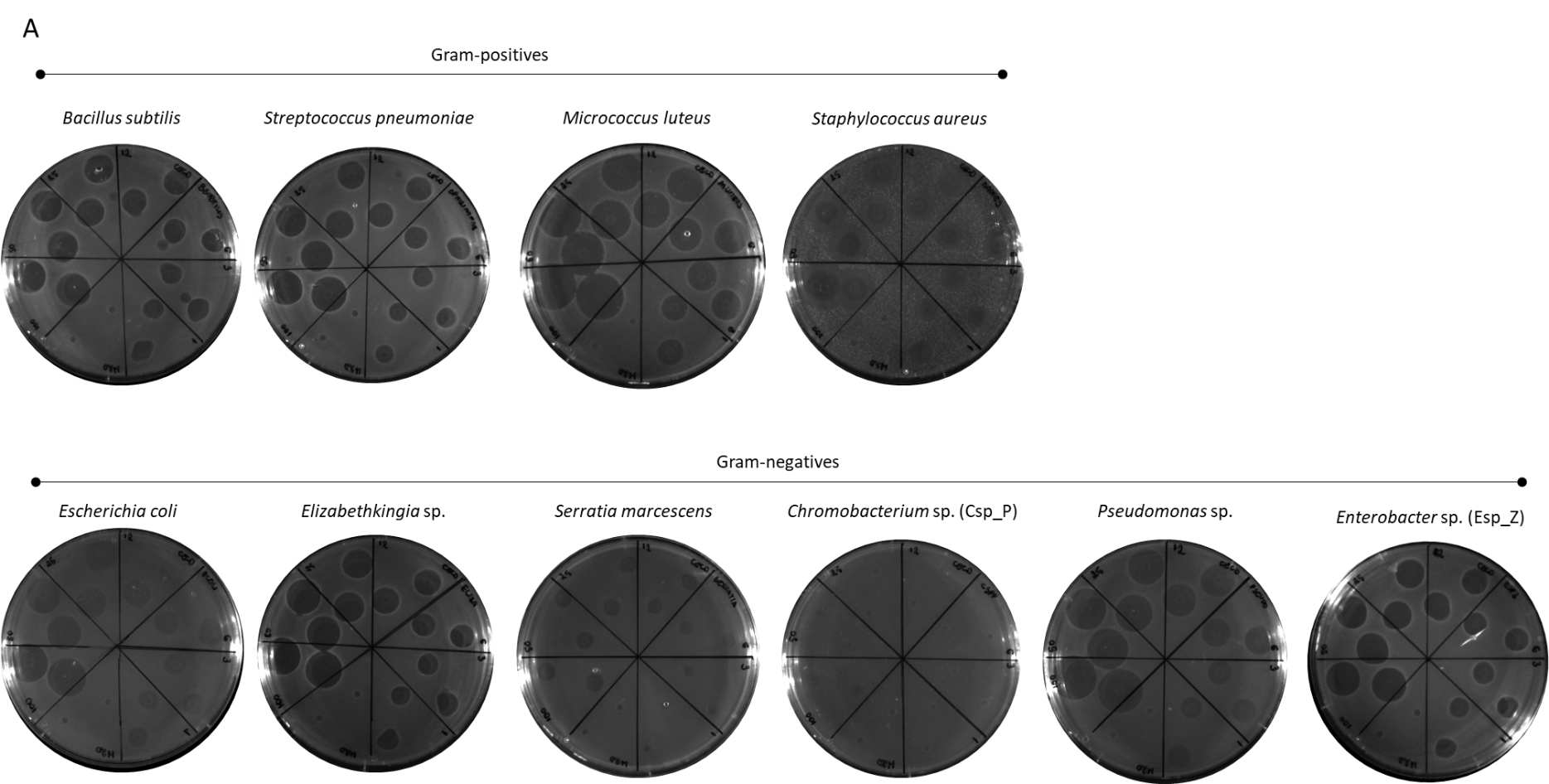


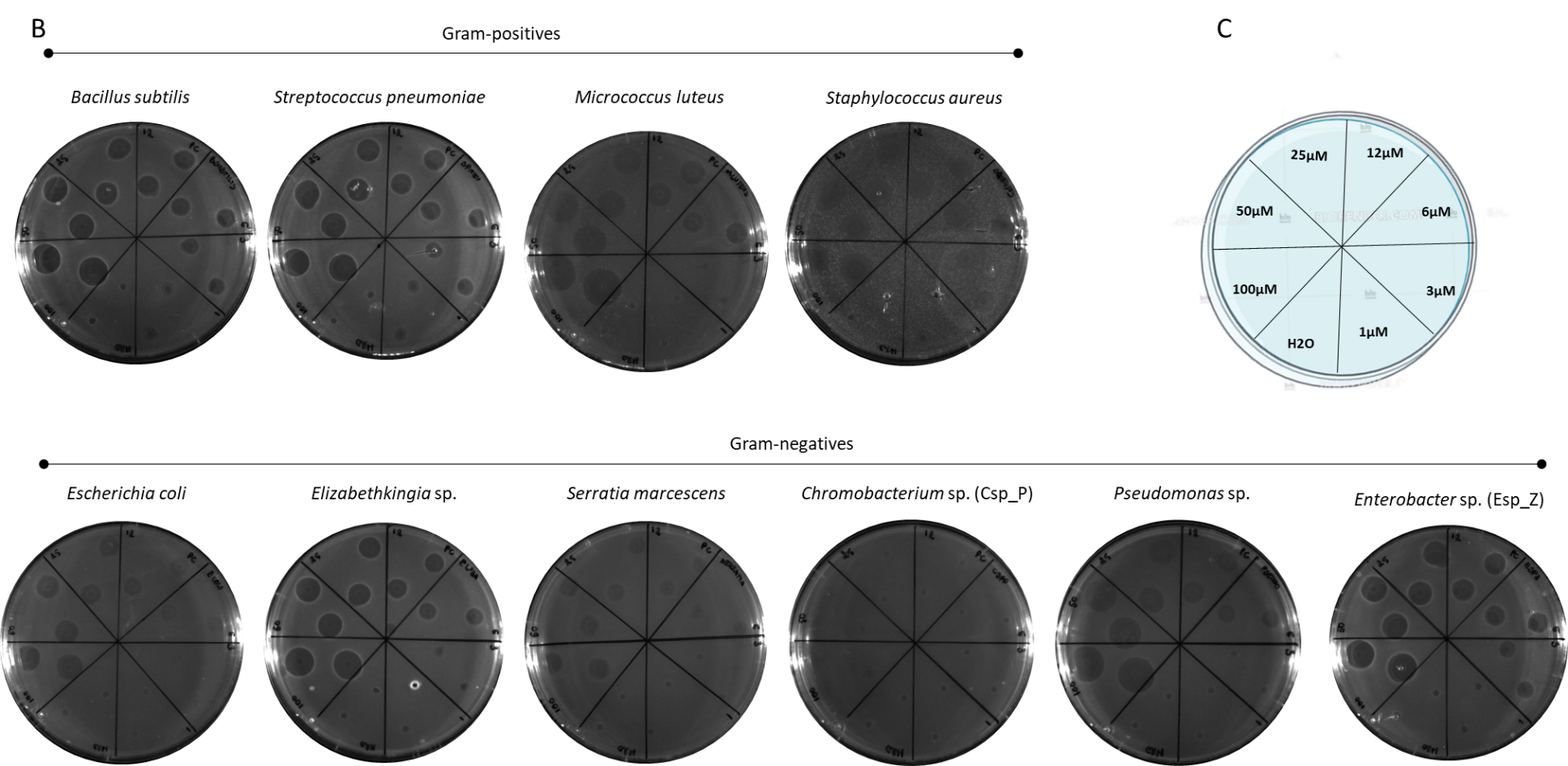

Supplement: S4 File — (A) Antibacterial activity of Anopheles gambiae cecropin D across the bacterial strains tested. (B) Antibacterial activity of positive control Hyalophora cecropia cecropin A peptide across the bacterial strains tested. (C) Schematic representation of peptide distribution on agar cultures for antimicrobial test. (DOCX) [file ppat.1012652.s004.docx]
