# Supplementary material for: A novel broad-spectrum antibacterial and anti-malarial Anopheles gambiae Cecropin promotes microbial clearance during pupation
Source: PLoS Pathog. 2024 Oct 23;20(10):e1012652. doi: 10.1371/journal.ppat.1012652 (PMC11554196; doi:10.1371/journal.ppat.1012652)
Supplement: S2 Table — (DOCX) [file ppat.1012652.s006.docx]

| **Table S2. List of primers used in this study** | | |  |
| --- | --- | --- | --- |
| **Gene Name** | **Primer name** | **Primer sequence (5'-3')** | **Reference** |
| *Primers for molecular cloning* | | | |
| Cecropin D | CecD-clo Fw | ACACTCGGGGTTTAAGCTGA | This study |
|  | CecD-clo Rv | TAATCTTCATCTCGCTGCGC |  |
| *Primers for Quantitative Analysis of Gene Expression (RT-qPCR)* | | | |
| Cecropin A | Cec1F-AG374 | CCAGAGACCAACCAACCACCAA | [1] |
|  | Cec1R-AG375 | GCACTGCCAGCACGACAAAGA |  |
| Cecropin B | Cec3F-AG376 | GTGCGCCGCGGTGGAAGT | [1] |
|  | Cec3R-AG377 | AATGACGGGCAGCGCTTTCTTAG |  |
| Cecropin C | CecC_qFw | GTACTGATGGCTGCGTTCCT | This study |
|  | CecC_qRv | GCTGCATTGGCTACTCGTC |  |
| Cecropin D | CecD qFw | CTGCTACTGTTCGGTGGTCA | This study |
|  | CecD qRv | GACCGTTCTTCTTGTCACGCA |  |
| RpS7 | RpS7 qFw | AGAACCAGCAGACCACCATC | [2] |
|  | RpS7 qRv | GCTGCAAACTTCGGCTATTC |  |
| *Primers for gene knock-down (RNAi)** | | | |
| Cecropin D | CecD-T7 Fw | taatacgactcactatagggTGAACGTATCGAAGCTGTTTG | This study |
|  | CecD-T7 Rv | taatacgactcactatagggCTGTATACCCTGCAGTACGGG |  |
| *Primers for Estimation of Total Bacterial Load* | | | |
| Universal 16S | 16S_Fw | ACTCCTACGGGAGGCAGCAG | [3] |
|  | 16S_Rv | GGACTACHVGGGTWTCTAAT |  |

*Sequence underlined represents the T7 promoter for *in vitro* RNA synthesis.

**References**

1. Frolet C, Thoma M, Blandin S, Hoffmann JA, Levashina EA. Boosting NF-κB-Dependent Basal Immunity of *Anopheles gambiae* Aborts Development of *Plasmodium berghei*. Immunity. 2006;25: 677–685. doi:10.1016/j.immuni.2006.08.019

2. Dong Y, Aguilar R, Xi Z, Warr E, Mongin E, Dimopoulos G. *Anopheles gambiae* immune responses to human and rodent *Plasmodium* parasite species. PLoS Pathog. 2006;2: 0513–0525. doi:10.1371/journal.ppat.0020052

3. Pike A, Dong Y, Dizaji NB, Gacita A, Mongodin EF, Dimopoulos G, et al. Changes in the microbiota cause genetically modified *Anopheles* to spread in a population. Science. 2017;357: 29. Available: https://www.science.org
