# Supplementary figures and images for: A novel broad-spectrum antibacterial and anti-malarial Anopheles gambiae Cecropin promotes microbial clearance during pupation
Source: PLoS Pathog. 2024 Oct 23;20(10):e1012652. doi: 10.1371/journal.ppat.1012652 (PMC11554196; doi:10.1371/journal.ppat.1012652)

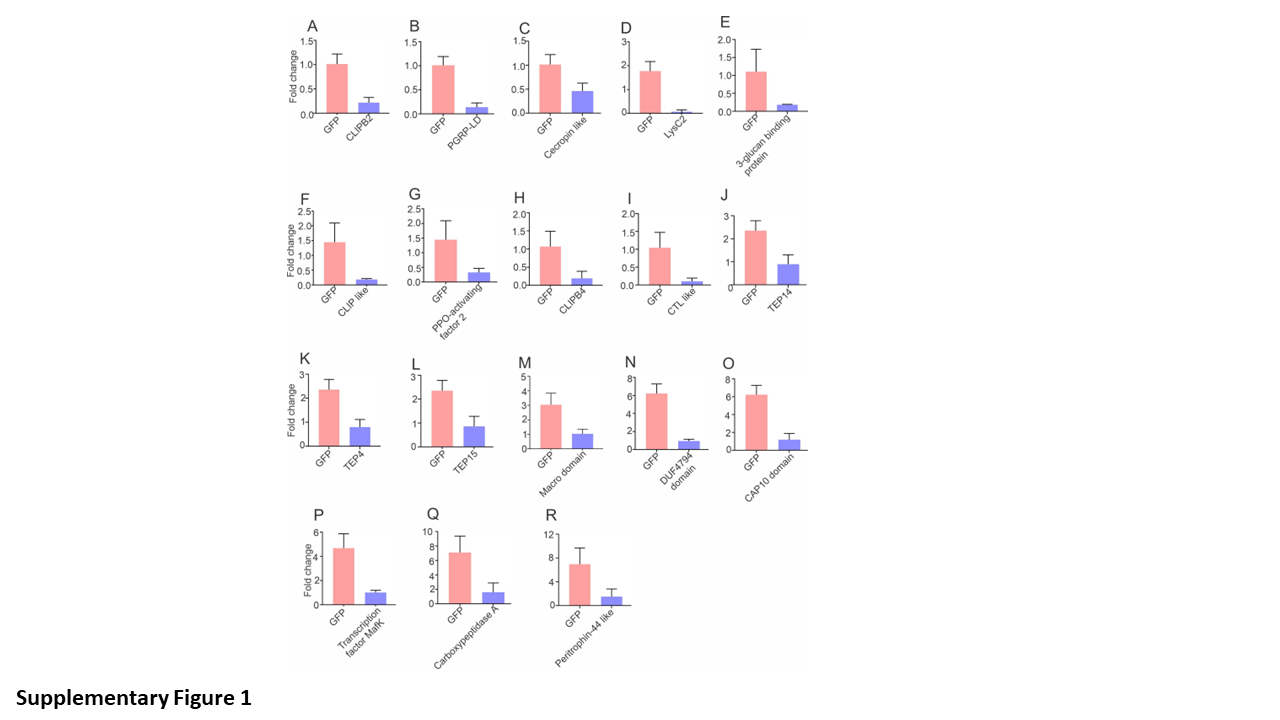

Supplement: S1 Fig — mRNA sequence of An. gambiae cecropin D and deduced amino acid sequence of its precursor peptide. The nucleotide sequence represents the consensus of fully sequenced clones generated by PCR. The arrows indicate the location and orientation of specific PCR primers used for RT-qPCR (continuous line), molecular cloning (square dashed lines) and in-vitro dsRNA transcription (round dashed lines). The predicted signal peptide is highlighted in italics, with an arrowhead marking the putative signal peptidase cleavage site. The stop codon is denoted by an asterisk. (TIF) [file ppat.1012652.s007.TIF]

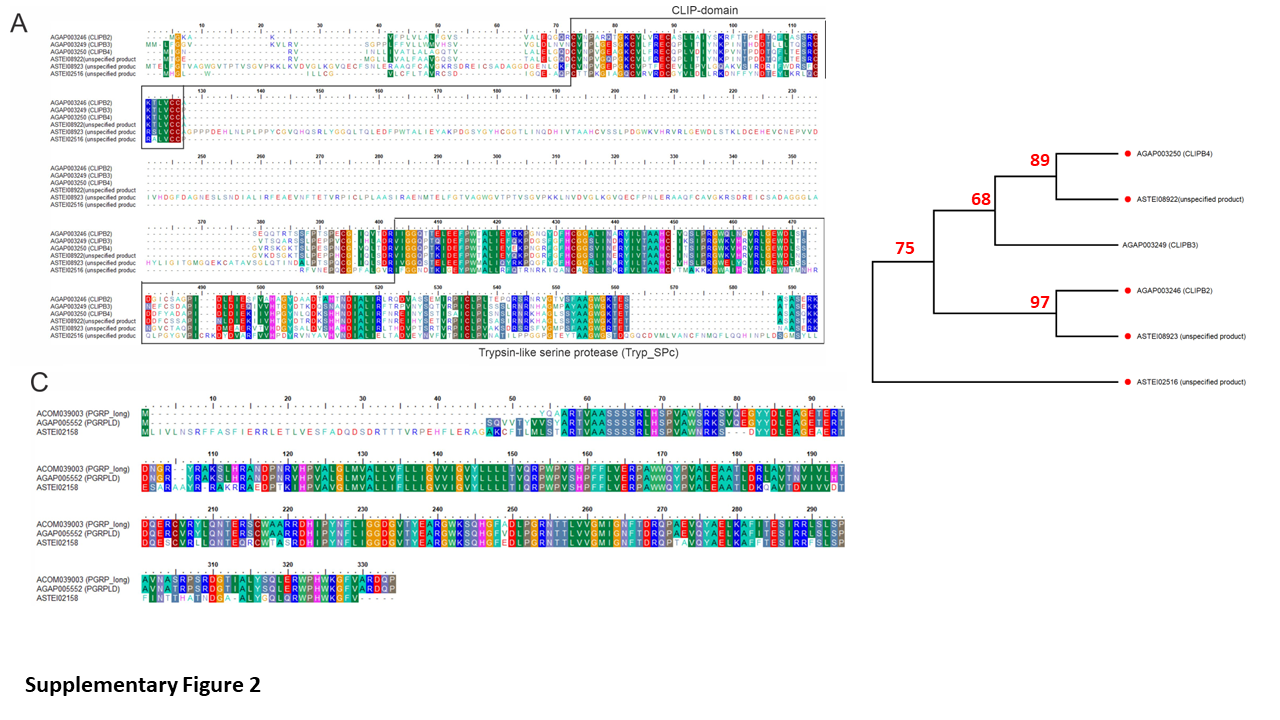

Supplement: S2 Fig — Amino acid sequence of cecropin mature peptides were analyzed for their general biochemical properties. Molecular weight, theoretical isoelectric point (pI) and grand average of hydropathy (GRAVY) values were retrieved using Expasy ProtParam tool. Net charge at physiological pH was obtained using a public server peptide calculator. (TIF) [file ppat.1012652.s008.TIF]

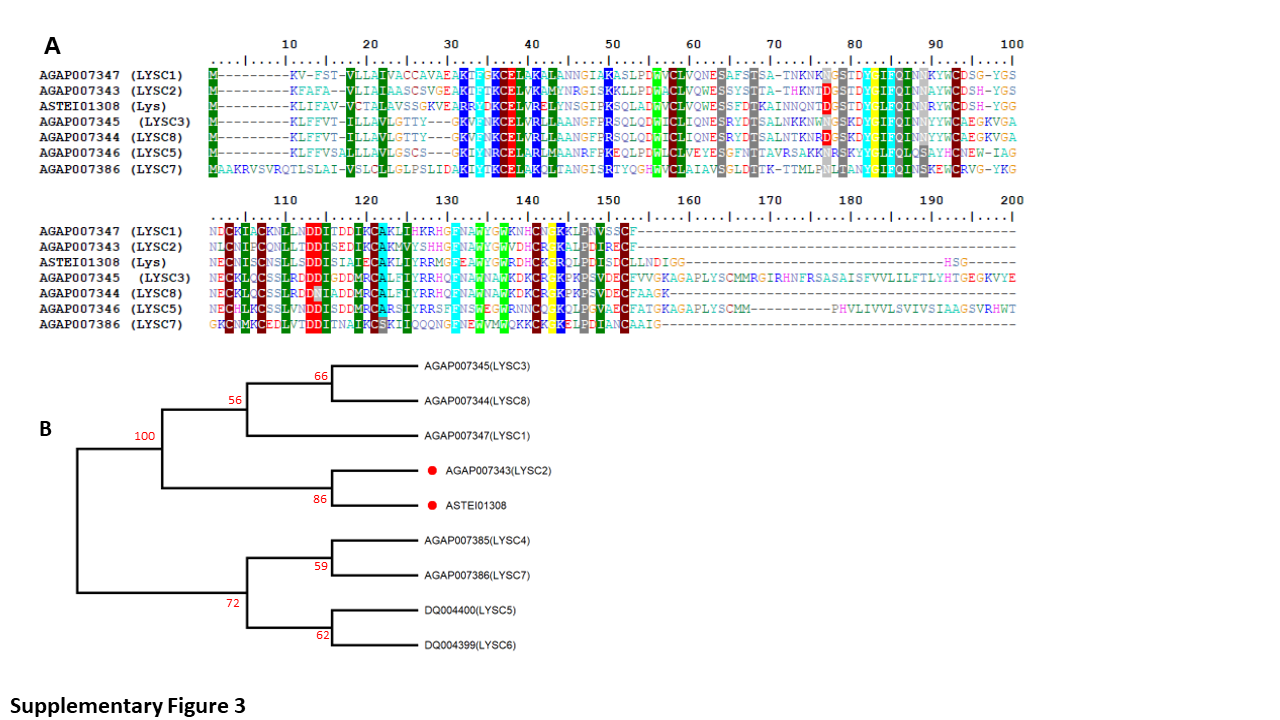

Supplement: S3 Fig — Amino acidic sequence of cecropin D was used to predict its tridimensional structure using template models available at I-TASSER server. Dark blue shows the predicted signal peptide, and light blue represents mature cecropin. Flexible hinge region is depicted in green, and the C-terminal cationic tail is shown in red. Lysine residue replacing typical tryptophan at position 2 is marked in orange. N- represents the amino-terminus. C- represents carboxy-terminus. (TIF) [file ppat.1012652.s009.TIF]

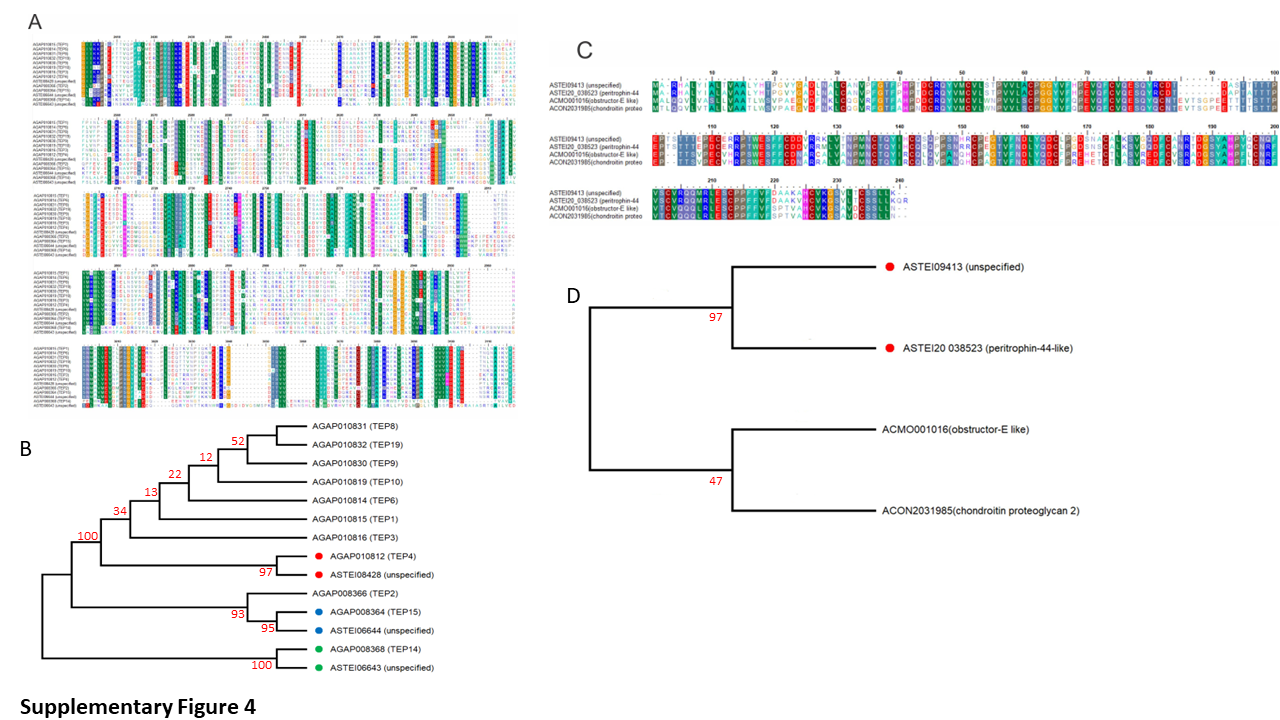

Supplement: S4 Fig — Amino acid sequences of cecropin mature peptides from diverse species of Anophelinae mosquitoes were aligned and conserved residues were identified as part of the molecular signature of each cecropin group. (TIF) [file ppat.1012652.s010.TIF]

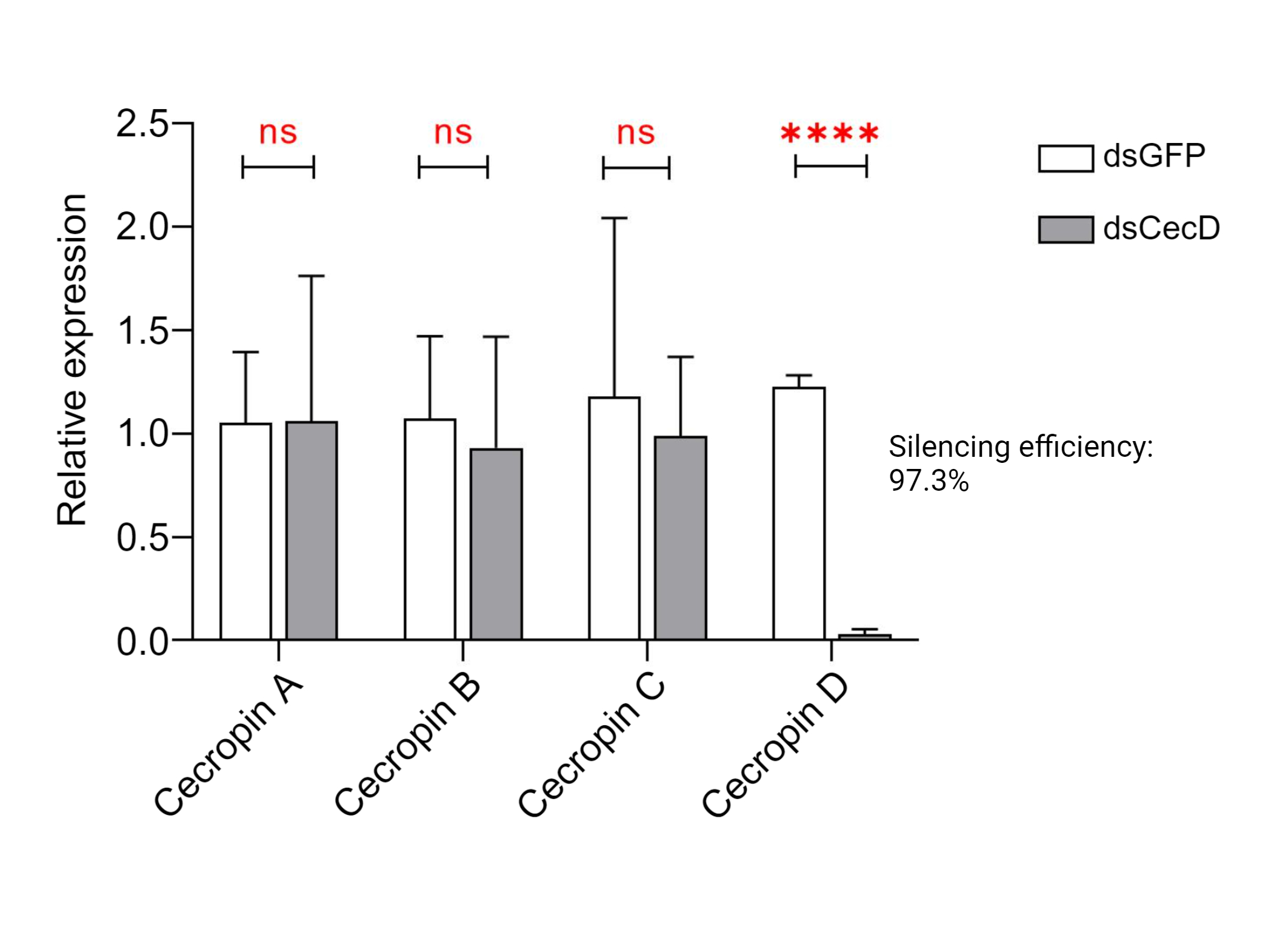

Supplement: S5 Fig — Fourth instar larvae were injected with either dsCecD or dsGFP, and transcript depletion of all An. gambiae cecropin genes was assessed by qRT-PCR at 24 hpi. Transcript levels of cecropin genes of dsCecD-injected larvae were measured relative to those of the dsGFP control, and An. gambiae RpS7 was used as an internal control. Data are shown as the mean of four biological replicates ± SD. Statistical significance was determined by unpaired t-test, and significance was defined as p < 0.05. ns: not significantly different. ****, p < 0.0001. (PNG) [file ppat.1012652.s011.png]
